# Genotypic Variability in Root Morphology in a Diverse Wheat Genotypes Under Drought and Low Phosphorus Stress

**DOI:** 10.3390/plants13233361

**Published:** 2024-11-29

**Authors:** Xin Li, Yinglong Chen, Yuzhou Xu, Haoyang Sun, Yamin Gao, Peng Yan, Qilong Song, Shiqing Li, Ai Zhan

**Affiliations:** 1 State Key Laboratory of Soil Erosion and Dryland Farming on the Loess Plateau, College of Natural Resources and Environment, Northwest A&F University, Yangling, Xianyang 712100, China; lixin7025@nwafu.edu.cn (X.L.); yinglong.chen@uwa.edu.au (Y.C.); xyz15138579869@163.com (Y.X.); gaoyamin@nwafu.edu.cn (Y.G.); 2 College of Resources and Environmental Sciences, Gansu Agricultural University, Lanzhou 730070, China; songql@nwafu.edu.cn; 3 The UWA Institute of Agriculture, School of Agriculture and Environment, The University of Western Australia, Perth, WA 6001, Australia; 4 College of Grassland Agriculture, Northwest A&F University, Yangling, Xianyang 712100, China; sun951hy@foxmail.com; 5 Baoji Academy of Agricultural Science, Baoji 722400, China; Yanpengwn@163.com

**Keywords:** winter wheat, low phosphorus stress, drought stress, genotypes, root traits, high-throughput phenotype system

## Abstract

Screening genotypes with optimal root traits presents a promising breeding strategy for enhancing adaptability to abiotic stresses and improving resource use efficiency. This study evaluated root traits of 100 winter wheat genotypes under four treatments: control (C), low phosphorus (LP), PEG-induced drought (D), and a combination of LP and drought (DLP), using a semi-hydroponic phenotyping platform. Significant variations in root traits were observed 65 days after transplanting, with over 80% of traits being significantly affected by drought, phosphorus, or their interactions. Biomass and phosphorus content decreased under LP and drought, while root length and diameter in deeper layers increased, especially under drought stress. Combined stress led to the most severe reductions in biomass, P-content, and leaf number. Phosphorus acquisition efficiency was positively correlated with root length but inversely related to stress tolerance. High heritability traits, such as root number, root length, maximum root depth, leaf number, and biomass, hold potential for breeding programs focused on environmental adaptation, resource efficiency, and yield improvement. The substantial genotypic variation in root morphology under stress conditions highlights the potential for breeding stress-resilient wheat genotypes. This finding lays a foundation for wheat-breeding initiatives aimed at developing genotypes better suited to prevailing environmental conditions.

## 1. Introduction

Wheat (*Triticum aestivum* L.) is one of the most widely grown crops in the world and holds a crucial social and economic position [1]. In China, winter wheat accounts for approximately 85% of both acreage and yield [2]. In the arid and semi-arid regions of northern China, the distribution of winter wheat is primarily constrained by the uneven distribution of rainfall, leading to an increased risk of drought-induced osmotic stress [3]. This stress can reduce water and nutrient utilization, ultimately limiting production [4]. Therefore, the selection of drought-resistant genotypes is crucial for increasing wheat yields and improving water use efficiency in this region.

Phosphorus (P) is a vital element for plant growth and development [5]. Over the past decades, a significant amount of P fertilizer has been applied to increase yields. However, more than 75% of the P fertilizer applied to the soil is not absorbed and utilized by the crop in the first growing season [6]. Low P utilization efficiency is attributed to various factors [7], with soil water status being one of the most significant [8]. Due to its poor mobility in the soil, P reaching the root surface is mainly through mass/bulk flow and diffusion, a process affected by soil moisture status. Drought stress decreases soil P availability, plant P uptake, and P utilization efficiency. Conversely, soil P availability also impacts plant water uptake and use efficiency. A comprehensive investigation into the combined effects of P and water on plant growth, as well as the efficiency of P and water, holds pragmatic significance for agricultural production.

Roots link plants to the soil environment and are the main site for nutrient and water absorption [9]. The high flexibility of root configurations is a key strategy for plants to cope with drought, soil infertility, and other edaphic stresses [10]. For example, in P-deficient environments, plant roots expand the effective surface area for P uptake by increasing root length and surface area [11], lateral root spreading [12], and the formation of specialized roots (e.g., cluster roots) [13]. In water-stressed environments, plants increase roots in the deep soil to access water stored in deeper soil [14]. Although root morphology is influenced by the environment, previous studies have shown that many root traits, such as deep and shallow root systems, number of seed roots, and lateral root density, are heritable [15,16]. Therefore, screening root configurations, especially across a large number of genotypes, is crucial for selecting root configurations adapted to different stressful environments. However, the process of screening is time-consuming, resource-intensive, and laborious, particularly in the context of root system screening. Although several root phenome studies have made some progress [17], high-throughput phenotyping studies of genotype–environment interactions are still limited. Chen et al. [18] developed a semi-hydroponic system, which allows for the quick and easy observation of root configurations, especially in relatively large numbers. Compared to traditional root research methods, this system allows for real-time monitoring of changes in two-dimensional root structure with minimal disturbance to plant growth and without the need for destructive root sampling. Additionally, it enables non-invasive, high-resolution observation of fine root structures, such as root hairs. This system was successfully applied to identify root phenotypes in maize (*Zea mays* L.) [19], soybean (*Glycine max* L.) [20], and wheat [21].

To mitigate the challenges posed by drought-induced osmotic stress and low phosphorus utilization in arid and semi-arid regions, an integrated approach to breeding and agronomic practices is needed. Recent advances in molecular breeding and precision agriculture offer promising avenues for developing drought-resistant and highly P-efficient genotypes that optimize resource use and productivity under environmental stress conditions. Moreover, previous studies predominantly focused on single-stress environments, such as water or phosphorus deficiency, while research addressing the combined effects of water and phosphorus stress often involved a limited number of test genotypes. In breeding programs adapted to suboptimal growing conditions, it is crucial to investigate the interactions among water status, nutrient uptake dynamics, and root morphological adaptations. Therefore, integrating both water and phosphorus conditions into large-scale observations and genotype screening holds significant value for breeding programs. In this study, the semi-hydroponic system was used to characterize the response of the root system of 100 wheat genotypes to P stress, drought stress, and combined P and drought stress. We hypothesized that (1) significant variation in root architecture traits exists among different wheat genotypes; (2) the responses of genotypes to different stress conditions could differ. The objectives of this study were to (i) analyze root traits at global and local levels in different winter wheat genotypes and their response to P and drought stresses; and (ii) explicitly explain the relationship of root trait variations with P acquisition and genotypic stress tolerance. This study will enhance our understanding of root trait variation in winter wheat under genotype–environment interactions and provide important information for breeding winter wheat for P and drought stress tolerance.

## 2. Results

### 2.1. Phenotypic Variation of Winter Wheat Genotypes

A total of 40 root traits and 10 shoot traits were analyzed (Table 1 and Table S1). Genotypes (G), phosphorus supply (P), water status (W), and their interactions showed significant differences in a number of parameters (*p* ≤ 0.05) (Table S2). Thirty-six root-related traits and all shoot-related traits were indicated as significantly different (*p* ≤ 0.05) among the 100 genotypes, with most of them even showing extreme significance (*p* ≤ 0.001). Forty-four and 45 parameters were significantly impacted (*p* ≤ 0.05) by P supply and water status. The interactions of P × W had a significant impact on 42 traits, while the G × P and G × W interactions had a significant effect on 24 and 20 traits, respectively (*p* ≤ 0.05). A total of 23 measured traits were affected by the G × P × W interaction (*p* ≤ 0.05).

**Figure 1:**
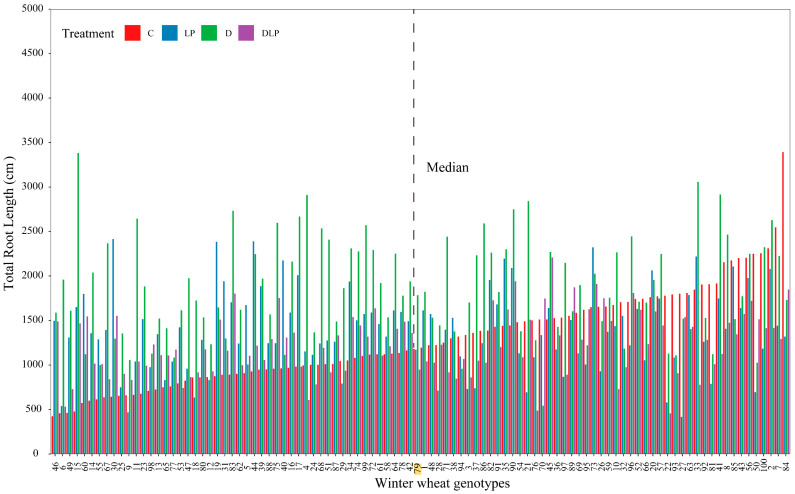
Genotypic variation in total root length of 100 winter wheat genotypes under the four treatments at 65 days after transplanting. The genotypes were sorted according to total root length in the control from smallest to largest. Different colors represent different root lengths for different treatments (control treatment, C; low phosphorus stress, LP; PEG-induced drought stress, D; combined drought and low phosphorus stress, DLP). The dashed line indicates the median value of total root length under control conditions. 1, Huazhang 166; 2, Fengdecunmai 21; 3, Yongminmai No. 1; 4, Pumai 116; 5, Yongfeng103; 6, Zhongzhimai No. 13; 7, Xinong 979; 8, Fengdeicunmai 22; 9, Hangyu 33; 10, Saidemai No. 8; 11, Womai 33; 12, Deyan 0516; 13, Baimai 312; 14, Huaimai 40; 15, Zhengmai 16; 16, Huaimai 226; 17, Xinong 556; 18, Lankao 298; 19, Xinmai 2111; 20, Hangyu 33; 21, Bainong 418; 22, Yongminmai No. 1; 23, Zhengmai 518; 24, Saidemai 601; 25, Xinong 0615; 26, Huaimai 920; 27, Zhongliang 91250; 28, Shannong 7064; 29, Hefeng No. 3; 30, Shunmai No. 11; 31, Minfeng 266; 32, Zhoumai 18; 33, Xuke No. 6; 34, Cunmai 633; 35, Bainong 558; 36, Luomai 26; 37, Ruiquanmai 32; 38, Qinnong 168; 39, Tainong 33; 40, Lunxuan 2000; 41, Lankaoai 6; 42, Yikemai No. 5; 43, Huaihe 12148; 44, Bonong No. 6; 45, Xinmai 21; 46, Yanzhan 4110; 47, Xunong No. 10; 48, Tunmai 257; 49, Xinong 501; 50, Longpingmai No. 3; 51, Cunmai 30; 52, Zhongmai 166; 53, Huaimai 1403; 54, Fumai 0808; 55, Xinong 20; 56, Jimai 44; 57, Yunong 168; 58, Baoliang No. 5; 59, Saidemai 601; 60, Suyu 0622; 61, Xiaoyan 22; 62, Baofeng 1530; 63, Shannong 116; 64, Pingan 0658; 65, Qinnong 578; 66, Quanmai31; 67, Saidemai No. 2; 68, Xinong 235; 69, Qinnong 28-6; 70, Zhongyu 1401; 71, Gaoke 115; 72, Luomai 906; 73, Huacheng 5157; 74, Bainong 207; 75, Huaihe 13068; 76, Taihemai No. 6; 77, Fengdecunmai 16; 78, Xinzhi No. 6; 79, Shunmai No. 10; 80, Wunong 68; 81, Xunong 029; 82, Xuke 718; 83, Jinxiu 21; 84, Qinnong 29; 85, Yumai 64; 86, Xinmai 38; 87, Qinmai 618; 88, Longke 1221; 89, Bainong 419; 90, Xiansheng 368; 91, Zhongyuan 20; 92, Huamai 304; 93, Tianmai 186; 94, Zhongmai 99; 95, Zhongzhongmai 27; 96, Yanfeng 168; 97, Huaimai 1196; 98, Zhongzhongmai 18; 99, Zhongzhimai No. 13; 100, Zhengmai 9023.

**Figure 2:**
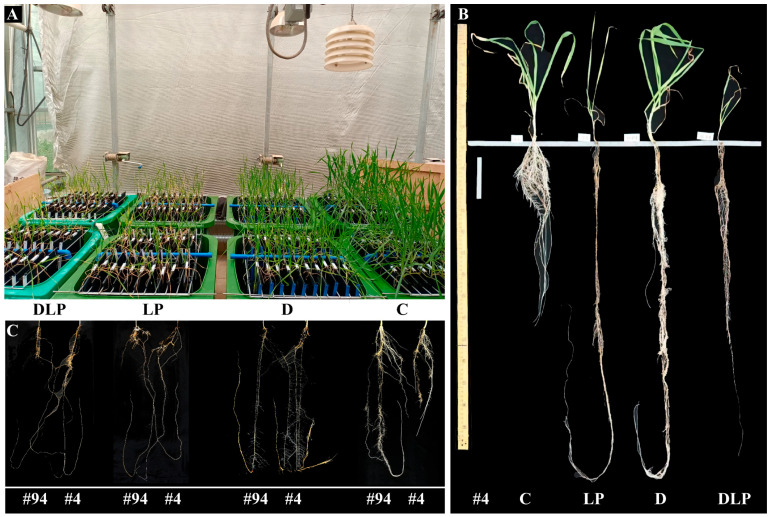
Winter wheat plants grown in a semi-hydroponic phenotypic platform (**A**). An example of one genotype (genotype #4) under four treatments (control treatment, C; low phosphorus stress, LP; PEG-induced drought stress, D; combined drought and low phosphorus stress, DLP) (**B**). An example of root systems of two genotypes (genotypes #94 and #4) under the four treatments (**C**). All photos were taken 65 days after transplanting.

At 65 DAT, most of the root traits and some shoot-related traits varied among the 100 genotypes (Table 2 and Table S3). Across all treatments, the coefficients of variation (CV) of 44, 39, 43, and 38 traits were all greater than 0.3. Relatively higher variation was found in root traits, including root length (RL), root volume (RV), root area (RA), and root length density (RLD). The mean values of RL, RV, RA, and RLD were significantly higher in the D treatment than in the other treatments (*p* ≤ 0.05). The longest RL was found in genotype #84 with 3393.53 cm under the C treatment (Table S4). The mean RL values were 1171.52 cm (C), 1763.34 cm (D), 1351.09 cm (LP), and 1213.64 cm (DLP). The mean RL of 100 genotypes under the four treatments is shown in Figure 1. The longest RL was about 8 times (C), 5.8 times (LP), 6.2 times (D), and 4.8 times (DLP) longer than the shortest RL, respectively. The maximum RL of genotype #15 in the D treatment was 2206.98 cm, which was only 476.97 cm in the C treatment. The maximum root depth (MRD), up to 120.97 cm, was found in genotype #4 under LP (Table S4, Figure 2). Root growth per day ranged from 0.43 cm (genotype #77, C) to 1.86 cm (genotype #4, LP) (Table S4). The mean values of average root diameter (RD) under the D, LP, and DLP were significantly greater than that under the C (*p* ≤ 0.05, Table 2). The ranges of RD were 0.10~0.48 mm (C), 0.12~0.56 (LP), 0.06~0.57 mm (D), and 0.11~0.57 mm (DLP), respectively (Table S4).

Root number (RN) showed significant differences (*p* ≤ 0.05) among the four treatments with mean values of 8.64 (C), 6.19 (LP), 4.91 (D), and 5.61 (DLP) per plant, respectively (Table S3). Specific root length (SRL) was significantly (*p* ≤ 0.05) higher in the D, LP, and DLP than in the C treatment (Table 2). Root length intensity (RLI) under the D treatment (28.46 cm·cm^−1^) was significantly higher than the C (24.88 cm·cm^−1^), while RLI in the LP (18.37 cm·cm^−1^) and DLP (17.83 cm·cm^−1^) treatments was significantly lower (*p* ≤ 0.05). Root tissue density (RTD) ranged from 0.02 to 1.49 mg·cm^−3^, with the DLP treatment having the lowest mean RTD among the four treatments and significantly lower than the C and D treatments. The mean values of plant height (SH), leaf number (LN), tiller number (TN), shoot growth rate (SGR), shoot dry weight (SDW), total dry weight (TDM), root phosphorus content (RP), root phosphorus concentration (RPC), shoot phosphorus content (SP), shoot phosphorus concentration (SPC), and total phosphorus content (TP) were significantly lower in three stress treatments than in the C treatment (Table 2 and Table S3) (*p* ≤ 0.05).

The local traits of the root system reflected the growth of the root segments at different depths and varied significantly among genotypes (CVs ≥ 0.3) (Table S3). Results showed that RL in the s3 and s4 root layers were significantly increased under the D, LP, and DLP treatments, and the distribution of RL in the s2 layer was significantly increased under the D treatment (*p* ≤ 0.05). RL in the s1 root layer decreased in the LP and DLP treatments (*p* ≤ 0.05). The mean values of RL_s1, RA_s1, RV_s1, and RLD_s1 under the LP and DLP treatments were significantly lower than those in the C and D treatments (*p* ≤ 0.05). RD_s1 in the DLP treatment was also significantly lower than the values of the C and D treatments (*p* ≤ 0.05). RL_s2, RA_s2, RV_s2, and RLD_s2 in the D treatment were significantly higher than those of the other three treatments except for RD_s2 (*p* ≤ 0.05). The mean values of RL, RA, RD, RV, and RLD in s3 and s4 root layers under the D, LP, and DLP treatments were significantly higher than those of the C (*p* ≤ 0.05). The CVs of the root length ratio (RLR_s1/sub) ranged from 0.67 to 1.34 under the four treatments, and the mean value in the C treatment was significantly higher than that of the three stress treatments (*p* ≤ 0.05).

### 2.2. Correlation Among Traits

**Figure 3:**
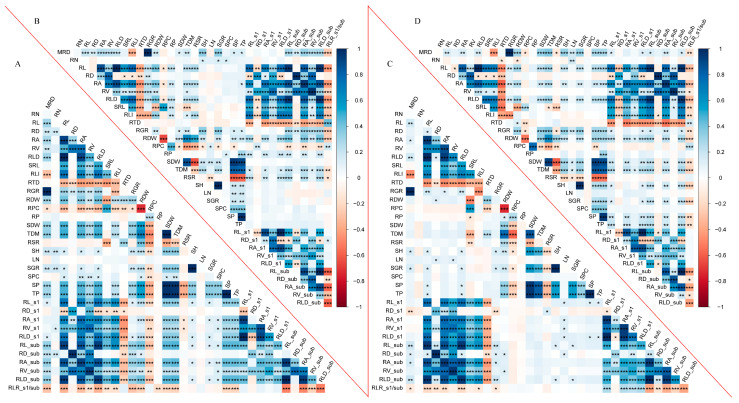
Pearson’s correlation analysis for root and shoot traits under four different treatments. (**A**) control treatment (C). (**B**) low phosphorus stress (LP). (**C**) PEG-induced drought stress (D). (**D**) combined drought and low phosphorus stress (DLP). Blue color indicates positive correlation and red color indicates negative correlation. The darkness of the color of the squares indicates the strength of the correlation. * *p* < 0.05, ** *p* < 0.01, *** *p* < 0.001. MRD, maximum root depth; RN, root number; RL, root length; RD, root diameter; RA, root area; RV, root volume; RLD, Root length density; SRL, specific root length; RLI, root length intensity; RTD, root tissue density; RGR, root growth rate; RDW, root dry weight; RPC, root phosphorus concentration; RP, root phosphorus content; SDW, shoot dry weight; TDM, total dry mass; RSR, root–shoot ratio; SH, shoot height; LN, leaf number; SGR, shoot growth rate; SPC, shoot phosphorus concentration; SP, shoot phosphorus content; TP, total phosphorus content; RL_s1, root length s1; RD_s1, root diameter s1; RA_s1, root area s1; RV_s1, root volume s1; RLD_s1, Root length density s1; RL_sub, root length in sub-layer; RD_sub, root diameter in sub-layer; RA_sub, root area in sub-layer; RV_sub, root volume in sub-layer; RLD_sub, Root length density in sub-root layer; RLR_s1/sub, Root length ratio.

A set of 35 traits, including 24 global traits and 11 root local traits, was used to establish the Pearson correlation matrix to identify correlations among the measured traits (Figure 3). Most of the traits showed strong correlations, and some of them even showed extremely strong correlations (*p* ≤ 0.001). The global and local traits, including RL, RA, and RV, were strongly associated with each other in all four treatments (*p* ≤ 0.001), except for RD (*p* ≤ 0.05). SP, RPC, and TP were associated with root morphology traits (global or local RL, RA, and RV) under the C treatment (*p* ≤ 0.01). SDW had a good correlation with RDW, RL, RL_s1, and RL_sub under the C treatment (Figure S1). Similarly, a linear function explained the variation of RL response to RDW in 79% and 63% under the C and LP treatments, respectively, whereas it was lower under the D and DLP treatments, 22% and 28%, respectively (Figure S2A).

The Pearson correlations between the rate of change (∆) in root length relative to the control for the three stress treatments and phosphorus acquisition efficiency (PAE) and stress tolerance score (STS) are shown in Table 3. There were significant negative correlations between STS and PAE, ΔRL, ΔRL_s1, and ΔRL_sub, whereas there were highly significant positive correlations between PAE and ΔRL, ΔRL_s1, and ΔRL_sub under the D, LP, and DLP treatments (*p* ≤ 0.01). In addition, ∆RD and ∆RD_sub showed a negative correlation with STS and a positive correlation with PAE under the LP treatment (*p* ≤ 0.05).

### 2.3. Determination of Root Trait Variability

**Figure 4:**
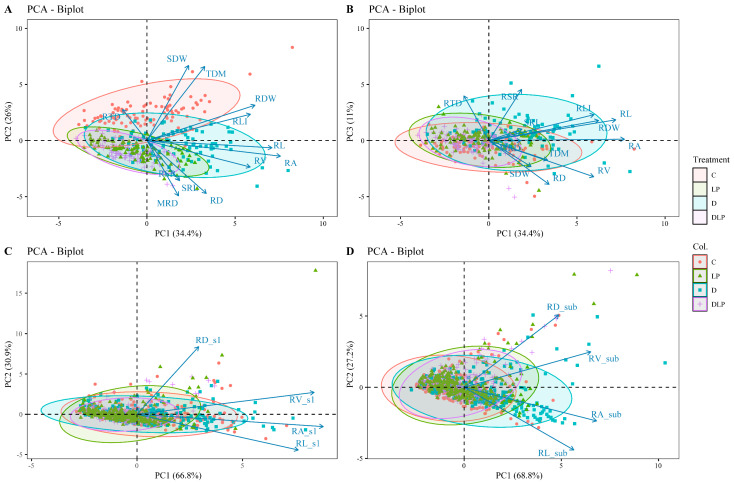
Principal component analysis of 100 winter wheat screening traits at 65 days after transplanting. (**A**) PC1 and PC2 explain 60.4% of the variance. (**B**) PC3 explain 11% of the variance. (**C**) Principal component analysis of 4 root traits of 0–20 cm (s1) root segments, with PC1 and PC2 explaining 97.7% of the variance. (**D**) Principal component analysis of 4 root traits of below 20 cm (sub) root segments, with PC1 and PC2 explaining 96% of the variance. Different colors represent different treatments. C, control treatment; LP, low phosphorus stress; D, PEG-induced drought stress; DLP, combined drought and low phosphorus stress. MRD, maximum root depth; RL, root length; RD, root diameter; RA, root area; RV, root volume; SRL, specific root length; RLI, root length intensity; RTD, root tissue density; RSR, root–shoot ratio; RDW, root dry weight; SDW, shoot dry weight; TDM, total dry mass; RL_s1, root length s1; RD_s1, root diameter s1; RA_s1, root area s1; RV_s1, root volume s1; RL_sub, root length in sub-layer; RD_sub, root diameter in sub-layer; RA_sub, root area in sub-layer; RV_sub, root volume in sub-layer.

Principal component analysis (PCA) was performed for (1) global traits (Table S5; Figure 4A,B), (2) 0–20 cm root layer (s1) traits (Figure 4C), and (3) below 20 cm root layer (sub) traits (Figure 4D). The first PCA on 12 selected global traits with CVs ≥ 0.3 (Table 2) in the control (Excludes RGR and RLD, which are calculated directly from MRD and RL) revealed four principal components (PCs) with eigenvalues > 1, which captured 81.2% of the variability across the tested genotypes. PC1 accounted for 34.4% of the total variability, consisting mostly of root-related traits such as RL, RA, RV, root length intensity (RLI), and RDW. PC2 accounted for 26.0% of the total variation primarily for MRD, RD, SDW, and TDM. PC3 accounted for 11.0% of the variation, mainly due to root tissue density (RTD) and root–shoot rate (RSR). Two principal components were extracted from the PCA for each of the four local traits in the two sections of the root system (top and sub-section) (Figure 4C,D). PC1 and PC2 in the top section (s1) accounted for 66.8% and 30.9%, respectively, while those in the sub-section accounted for 68.8% and 27.2%.

**Figure 5:**
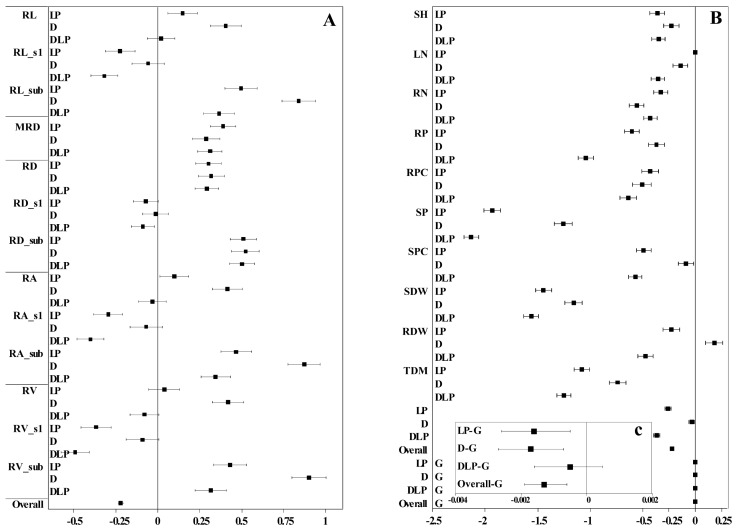
Forest plot of plant traits under different stress treatments. (**A**) The effect values of root traits; (**B**) the effect values of wheat global traits; (**C**) the effect values of the genotype. Overall means of effect size and 95% confidence intervals were given; the stress treatment effects were interpreted when the zero line is not crossed by the confidence intervals. Where C, control treatment; LP, low phosphorus stress; D, PEG-induced drought stress; DLP, combined drought and low phosphorus stress. RL, root length; RL_s1, root length s1; RL_sub, root length in sub-layer; MRD, maximum root depth; RD, root diameter; RD_s1, root diameter s1; RD_sub, root diameter in sub-layer; RA, root area; RA_s1, root area s1; RA_sub, root area in sub-layer; RV, root volume; RV_s1, root volume s1; RV_sub, root volume in sub-layer; SH, shoot height; LN, leaf number; RN, root number; RP, root phosphorus content; RPC, root phosphorus concentration; SP, shoot phosphorus content; SPC, shoot phosphorus concentration; SDW, shoot dry weight; RDW, root dry weight; TDM, total dry mass.

Drought and low phosphorus treatments showed an overall negative effect on plant traits with a total effect value of −0.22, and the confidence interval ranged from −0.20 to −0.24 (Figure 5). Among them, the overall negative effect of the DLP (−0.37) and LP (−0.26) treatments on plant traits was greater than that of the D treatment (−0.03). All three stress treatments had the largest negative effects on SP, SDW, and TDM, whereas positive effects were exhibited on MRD and root traits in the sub-layer. In addition, the D treatment had the greatest positive effect on RV_sub (0.90), RA_sub (0.87), and RL_sub (0.84).

### 2.4. Genotype Homogeneous Grouping Based on Root Trait Variation

**Figure 6:**
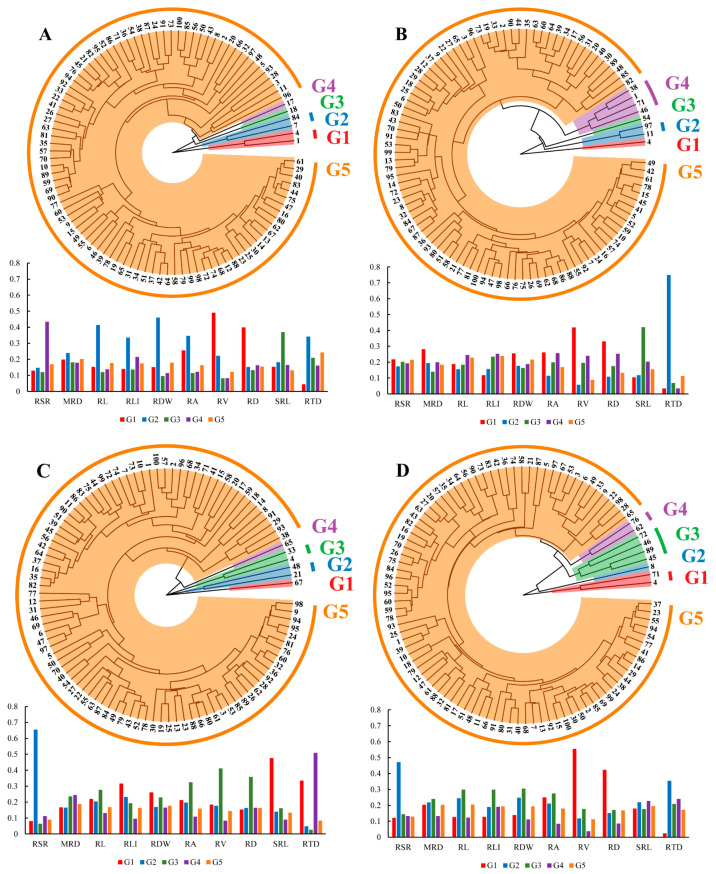
Agglomerative hierarchical clustering (AHC) dendrograms were constructed using the average linkage method for the 10 selected root global traits (same root traits as in PCA) with CV ≥ 0.3 using Euclidean distance as the interval measure. Bar charts showed the distribution of key root traits within clusters. (**A**) control treatment (C); (**B**) low phosphorus stress (LP); (**C**) PEG-induced drought stress (D). (**D**) combined drought and low phosphorus stress (DLP). Different colors represent different groupings only. 1, Huazhang 166; 2, Fengdecunmai 21; 3, Yongminmai No. 1; 4, Pumai 116; 5, Yongfeng103; 6, Zhongzhimai No. 13; 7, Xinong 979; 8, Fengdeicunmai 22; 9, Hangyu 33; 10, Saidemai No. 8; 11, Womai 33; 12, Deyan 0516; 13, Baimai 312; 14, Huaimai 40; 15, Zhengmai 16; 16, Huaimai 226; 17, Xinong 556; 18, Lankao 298; 19, Xinmai 2111; 20, Hangyu 33; 21, Bainong 418; 22, Yongminmai No. 1; 23, Zhengmai 518; 24, Saidemai 601; 25, Xinong 0615; 26, Huaimai 920; 27, Zhongliang 91250; 28, Shannong 7064; 29, Hefeng No. 3; 30, Shunmai No. 11; 31, Minfeng 266; 32, Zhoumai 18; 33, Xuke No. 6; 34, Cunmai 633; 35, Bainong 558; 36, Luomai 26; 37, Ruiquanmai 32; 38, Qinnong 168; 39, Tainong 33; 40, Lunxuan 2000; 41, Lankaoai 6; 42, Yikemai No. 5; 43, Huaihe 12148; 44, Bonong No. 6; 45, Xinmai 21; 46, Yanzhan 4110; 47, Xunong No. 10; 48, Tunmai 257; 49, Xinong 501; 50, Longpingmai No. 3; 51, Cunmai 30; 52, Zhongmai 166; 53, Huaimai 1403; 54, Fumai 0808; 55, Xinong 20; 56, Jimai 44; 57, Yunong 168; 58, Baoliang No. 5; 59, Saidemai 601; 60, Suyu 0622; 61, Xiaoyan 22; 62, Baofeng 1530; 63, Shannong 116; 64, Pingan 0658; 65, Qinnong 578; 66, Quanmai31; 67, Saidemai No. 2; 68, Xinong 235; 69, Qinnong 28-6; 70, Zhongyu 1401; 71, Gaoke 115; 72, Luomai 906; 73, Huacheng 5157; 74, Bainong 207; 75, Huaihe 13068; 76, Taihemai No. 6; 77, Fengdecunmai 16; 78, Xinzhi No. 6; 79, Shunmai No. 10; 80, Wunong 68; 81, Xunong 029; 82, Xuke 718; 83, Jinxiu 21; 84, Qinnong 29; 85, Yumai 64; 86, Xinmai 38; 87, Qinmai 618; 88, Longke 1221; 89, Bainong 419; 90, Xiansheng 368; 91, Zhongyuan 20; 92, Huamai 304; 93, Tianmai 186; 94, Zhongmai 99; 95, Zhongzhongmai 27; 96, Yanfeng 168; 97, Huaimai 1196; 98, Zhongzhongmai 18; 99, Zhongzhimai No. 13; 100, Zhengmai 9023.

Ten root global traits with CV ≥ 0.3 (the same as the root traits in PCA) were selected, and the Euclidean distance was used as the interval measure to construct the hierarchical clustering (AHC) tree by the average linkage method (Figure 6). The results showed a large trait diversity among 100 winter wheat genotypes under different treatments. Clustering divided the 100 genotypes into five groups under each treatment. Of these, more than 90% of the genotypes were classified into one group, which was further separated into several subgroups. In the C treatment (Figure 6A), the first group (G1) contained two genotypes, including genotypes #1 and #4 with the highest RV and RD; the second group (G2) consisted of genotypes #7 and #84, which had high levels of RDW, RL, RA, RLI and RTD; and the third (G3) and fourth (G4) groups each contained one genotype, #18 (highest SRL) and #17 (highest RSR), respectively. The six genotypes were separated into four groups in the D treatment (Figure 6C). Under the LP treatment, there were eight genotypes divided into four groups: G1 and G3 each contained one genotype, genotypes #4 and #7, respectively; G2 consisted of genotypes #11 and #97, and the remaining genotypes were divided into group G4. And nine genotypes from the DLP treatment were separated into four groups (Figure 6D).

One hundred winter wheat genotypes under D, LP, and DLP were divided into five groups based on the stress tolerance score (STS) (Figures S3 and S4). Genotype #84, with the highest STS (Table S6), was placed in Group I; genotypes #7 and #57 were placed in Group II, with STS rankings of second and third; genotype #17, with the lowest STS, was placed in Group III; and intermediate genotypes were placed in Groups IV (including 43 genotypes) and V (including 53 genotypes), respectively, and further subdivided into numerous subgroups.

## 3. Discussion

### 3.1. Genetic Variation of Winter Wheat Root Traits

Roots are directly involved in the competition and utilization of nutrients and water, playing a crucial role in plant morphogenesis and biomass accumulation [22]. Consequently, there has been increasing attention paid to root phenotypic identification in crop breeding [23]. Chen et al. categorized root traits as “performance traits” and “functional traits” [21]. Directly measured traits such as root length (RL), root volume (RV), surface area (RA), and root dry weight (RDW) are termed performance traits, while functional traits indirectly affect plant fitness by influencing growth, reproduction, and survival [24]. Previous studies on winter wheat have reported that root performance traits can directly characterize the size of the plant root system [25] and are closely related to below-ground competition, nutrient and water uptake, and overall production [26,27]. For instance, in wheat, deeper and larger roots with more root hairs were shown to improve root access to water from deeper soil layers [28]. In wild tobacco (*Nicotiana attenuata* L.), a more developed shallow root system is more conducive to rapid uptake of shallow water and nutrient foraging by the plant [29]. Rice genotypes with vigorous root systems are more conducive to canopy development and yield improvement [30]. However, root size varies significantly between species and even among different varieties of the same species. Results in the present study also indicated that root traits related to root size, including RL, RA, RV, and RDW, vary widely among genotypes, and the selection of optimal root traits is crucial for ensuring genetic gain and sustainable wheat production [26].

The root system is dynamic and highly plastic, allowing it to make decisions about its growth and development in response to the environment around it [26,31]. As genotypes respond differently to drought and low P, variations in their root system conformation produce more interesting phenomena. These variations are key to understanding plant strategies for coping with different environments. Root traits provide a good basis for understanding the distribution of root length and root distribution characteristics [32]. Differences in root distribution and growing patterns are more specifically characterized by localized roots at different depths. In the present study, root local traits across all treatments showed large variation (CV ≥ 0.25) among genotypes. Studies have shown that plants with extensive shallow root systems are more efficient at uptaking nutrients in the topsoil layer, especially low-mobility nutrients mainly located in the topsoil layer, such as P [33,34]. In arid and semi-arid areas, plants with shallow root systems can also absorb water more quickly when it rains, aiding plants in surviving droughts [35]. On the other hand, plants with large sublayer root systems can explore and access nutrients and water from deep soil layers [36], which is beneficial for plant resistance to drought stress. Although many characteristics of the root system are influenced by the environment, previous studies have pointed out that some root traits, such as deep roots, root length, root number, and lateral root density, are heritable traits [16,37]. In this study, we also found that more than half of the measured traits had broad-sense heritability greater than 50% (Table S7). It was suggested that it is possible to screen crops for root configurations that are compatible with specific environments. For example, in low P soils, soybeans and maize with shallow root systems are selected to have higher phosphorus use efficiency and yield [38]. In drought conditions, bread wheat with deep root systems has greater drought tolerance [39]. Some agro-ecosystems also utilize intercropping of the strong deep roots of grasses with the taproot system of legumes occupying different spatial ecological niches to achieve efficient and sustainable use of land resources [40].

### 3.2. Variation of Winter Wheat Shoot and Root Traits to Drought and Phosphorus Stress

In breeding programs, aside from understanding the genetic differences in the traits of the plants themselves, comprehending their resistance traits is crucial for variety selection and for identifying key genes for stress tolerance [41]. In this study, more than 80% of the measured traits exhibited highly significant differences in response to LP, D, and the interactions of LP and D, indicating that the LP and D stress treatments significantly affected winter wheat shoot and root traits. Previous studies have reported delayed shoot growth, reduced biomass accumulation, and P uptake when plants are exposed to drought or low P stress [42,43], which aligns with the findings of the present study. Shoot growth traits such as SDW, SH, LN, TN, RSR, and P content and concentration in the LP, D, and DLP treatments were significantly lower than in the C treatments (Table 2 and Table S3). The LP, D, and DLP treatments had the greatest negative effect on SP (Figure 5). MRD, RL, RA, RV, and RSR were significantly higher under LP, D, and DLP treatments compared to the C, suggesting that both D and LP promote an increase in the extent of root extension.

**Figure 7:**
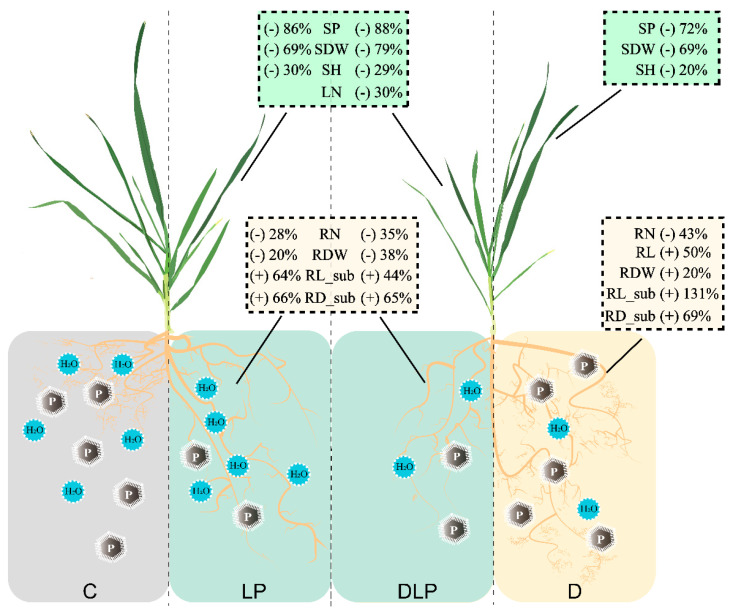
Schematic representation of wheat plant variation under different treatments in this study. Where C, control treatment; LP, low phosphorus stress; D, PEG-induced drought stress; DLP, combined drought and low phosphorus stress. SP, shoot phosphorus content; SDW, shoot dry weight; SH, shoot height; LN, leaf number; RN, root number; RDW, root dry weight; RL, root length; RL_sub, root length in sub-layer; RD_sub, root diameter in sub-layer.

Drought stress often leads to alterations in root traits, such as increased RL and RLD, to optimize water uptake [36]. In this study, RL, RA, RV, RDW, RLD, RLI, and RSR under the D treatment were significantly higher than those in the rest treatments (Table 2 and Table S3), suggesting that a certain degree of D stress induced a more extensive root system in winter wheat. These results are consistent with previous studies [44]. The greatest positive effect was observed in the D treatment on the root in the sublayer (RV_sub, RA_sub, and RL_sub, Figure 5), indicating that winter wheat adapts to water shortage by increasing the growth of deep roots, which aligns with earlier studies [45,46]. Additionally, the D treatment increased the RDW of winter wheat, whereas both the LP and DLP treatments decreased the RDW of winter wheat (Figure 7). This might be the result of the fact that under a single moderate drought treatment, the plant had enough nutrients to support the expansion of the root system to withstand drought stress.

Under low phosphorus conditions, plants may exhibit modifications in root configurations, such as enhanced lateral root development, to improve P acquisition efficiency [47]. Specific root length (SRL) is the root length per unit mass, and higher SRL values indicate finer roots and a root system with a larger absorptive area, which is important for improving the uptake of low-mobility nutrients, like P [48]. In this study, SRL in the P deficiency treatment (LP and DLP) was significantly higher than in the C, and this was mainly attributed to the decreased RDW and non-changed RL (Table 2). Under low P stress, plants increase RL by reducing secondary root growth and metabolic costs to the root system, thereby improving P absorption and above-ground biomass [49]. The maximum positive effect on RD_sub was observed in the LP and DLP treatments (respectively, 0.51 and 0.50) (Figure 5). Low phosphorus stress promoted an increase in root diameter, consistent with the results of [50]. Ref. [51] in *Lactuca* mentioned that genotypes with larger root apical diameters possessed relatively increased phosphorus utilization efficiency under low phosphorus. The present study also supports this result (Table 3). Compared with LP and D treatment groups, the DLP treatment inhibited lateral and vertical root growth by reducing the concentration and content of phosphorus in roots and shoots, ultimately causing a significant decrease in plant biomass (Figure 5, Table 2). This may be due to the fact that wheat maintains shoot growth by abandoning part of the root growth when faced with intense or complex stresses [52,53]. These results may indicate that some genotypes that are resistant to one factor may be limited by another factor. For example, genotype #4 showed stress tolerance under the D treatment (Figure 2B); under the LP treatment, it still increased root extension capture area and stress tolerance. However, under the DLP treatment, there was not enough energy to maintain root elongation and stem growth due to dual water-phosphorus deficiency. Previous studies reported that a positive correlation exists between total root length, root biomass, and aboveground biomass accumulation under nutrient–water-sufficient conditions [21]. However, these relationships were weakened or masked under stress conditions in the present study. This was also confirmed in the broad-sense heritability estimate (Table S7). Managing the interaction between environmentally induced variation and genetic variation is essential for effective plant breeding strategies.

Stress-induced root variation provides more possibilities for breeding programs that utilize root traits. According to the study of [54], P absorbed by plants is mainly determined by root length. In this study, it was observed that the phosphorus uptake efficiency of winter wheat was significantly positively correlated with the total root length increment (Δ) under three stress conditions, and inversely correlated with the STS (Table 3). This result may make it possible in the future to assess the PAE and stress tolerance indices of different genotypes in specific environments by measuring root phenotypes such as RL. Using their strong correlation and combining them with soil environmental characteristics to build models. The phosphorus use efficiency and stress tolerance of genotypes in specific environments could be assessed or predicted without measuring phosphorus content and uptake efficiency, reducing the cost of genotype selection in the breeding process.

### 3.3. Genotype Selection Based on Root Trait Properties for Breeding Programs

Global climate change poses numerous challenges to the world’s ecosystems [55]. Enhancing crop resource utilization efficiency and adapting to resource-saving farming systems are the primary objectives of modern crop breeding programs [56]. Due to the great potential in enhancing plant adaptation to different environmental stresses, considering root morphological configuration in future breeding work is important [57]. To optimize P, water, and N acquisition, the ideotype root architecture of ‘topsoil foraging’ and “Steep, Cheep, and Deep” were put forward by the group of Lynch [12,33,36], and have guided the development of maize, bean, and soybean cultivars with enhanced P, water, and N acquisition [58,59,60].

Crop functional genomics has entered the era of big data and high throughput, but research on root traits lags behind that on aboveground traits due to difficulties in root sampling and detection methods [17]. In the present study, an efficient semi-hydroponic phenotyping system was used to analyze the shoot and root response of 100 winter wheat varieties to drought, low P, and the interactions of drought and low P. Among the tested genotypes, Qinnong 29 (#84) and Xinong 979 (#7) exhibited higher STS under drought and low P treatments, suggesting their potential as candidates for drought and low-P breeding programs (Table S8). Additionally, Pumai 116 (#4) demonstrated significant root architectural changes under stress conditions, including higher MRD, RV, and RD, providing valuable insights for selecting drought-resistant and P-efficient winter wheat genotypes. Although this study was carried out under a human-controlled environment, which is very different from the variable and complex field environment. It should not be ignored that semi-hydroponic systems are ideal tools for efficiently evaluating deep root traits of a large number of genotypes in a relatively small space [18]. The study of water and nutrient stress under indoor controlled conditions effectively avoids the interference caused by complex environmental factors. The semi-hydroponic system allows real-time observation of root growth throughout the experiment with minimal impact on plant development. Additionally, the collection of root trait data has almost no loss, which greatly ensures the reliability of plant root evaluation. Furthermore, limited by hydroponics, PEG was used to simulate drought stress. One must acknowledge that the absence of certain physicochemical and biological components in PEG-based simulations may result in a partial representation of the multifaceted interactions inherent in actual drought stress environments. Although some studies have pointed out that PEG cannot truly reflect the water stress environment [61], numerous studies have explored the application of PEG in eliciting drought-like responses in plants, thereby serving as an invaluable tool in elucidating the underlying physiological and molecular mechanisms associated with water deprivation [62,63,64]. In summary, further studies will be carried out in the field to verify the greenhouse results.

## 4. Materials and Methods

### 4.1. Plant Materials and Experimental Design

The experiment was conducted in a temperature-controlled glasshouse at Northwest Agriculture and Forestry University (NWAFU), Yangling (34°160′ N, 108°4′ E), China, from January to March 2021, using a semi-hydroponic phenotyping system [18] (Figure 2). Shaanxi Province is one of the major winter wheat production areas in China, and a total of 100 wheat genotypes were selected for regional testing in Shaanxi Province in this study. Seeds for testing were provided by the Baoji Institute of Agricultural Science (Table S8).

Each semi-hydroponic phenotyping system comprised a 240-litre bin, 20 growth units consisting of transparent acrylic panels (250 × 500 mm, 4 mm thick) wrapped in black cotton, pumps, watering systems, and support frames. For specific descriptions, refer to [21] (Figure 2). Each bin was filled with 30 L of a nutritional solution, slightly modified from Hoagland’s nutrient solution, and consisting of the following concentrations (µM): K (1220), S (1802), Ca (600), Mg (200), Cu (0.2), Zn (0.75), Mn (0.75), B (5), Co (0.2), Na (0.06), Mo (0.03), Fe (40), and N (1000).

The experiment included four treatments: (1) the control group with sufficient P (300 μM KH_2_PO_4_) supply (C); (2) low P supply (3 μM KH_2_PO_4_, referred to as LP); (3) polyethylene glycol-6000-induced drought stress (PEG-6000 15%, *w*/*w*) (D); (4) combined drought and low P stress (DLP). Potassium chloride (KCl) was supplied for K compensation in low P treatments. Seeds of the 100 genotypes were surface sterilized in 10% H_2_O_2_ for 10 min, rinsed, and imbibed for 24 h in aerated 1 mM CaSO_4_, then placed in a dark and humid environment at 28 ± 1 °C for 4 days. Seedlings were transplanted to the growth plates of the semi-hydroponic system. Each growth plate was randomly transplanted with two plants of different genotypes. Four replicates were set up for each genotype, staggered for three days between replicates, and sowing time was considered a block effect. All bins were arranged in a randomized block group design.

The average daily temperature during the trial period was approximately 20 / 10 °C (day/night). The timing of water supply was controlled by a timer. Nutrient solution was supplied regularly for the first 7 days after transplanting (DAT) to enable the plants to quickly acclimate to the growing conditions. Each bin was randomly relocated once a week, and the nutrient solution was fully replenished once a week.

### 4.2. Sampling

Plants were harvested at 65 DAT. One day before harvest, shoot height, leaf number, and tiller number per plant were measured manually. At harvest, growth plates were taken out from the bin and placed on a flat sheet with a black background. Root systems were imaged using a digital camera (Sony LICE-7, Sony Corporation, Tokyo, Japan). The maximum root depth and root number (including the primary root and seminal roots from the seed) of each plant were recorded manually at harvest. Then, shoots were cut from the roots, oven-dried at 70 °C for 72 h, and ground for total-P analysis after biomass determination. To investigate the growth and stress response characteristics of winter wheat roots, it is necessary to analyze and identify root samples at different depths. Subsamples of roots were collected by cutting the root system (every 20 cm along the glass sheet) into 20 cm sections starting from the base and stored in a 4 °C refrigerator until scanning.

Root subsamples were scanned at 300 dpi using a flatbed scanner (Perfection V700 Photo; Epson, Long Beach, CA, USA), and root images were analyzed using the image-processing software WinRhizo Pro (v2009, Regent Instruments, Montreal, Québec City, QC, Canada). In order to compare root distribution strategies of different genotypes of wheat with deep roots or shallow roots, the initial 20 cm root segment (s1) and the root segment below 20 cm to the root tip were considered the ‘top-root layer’ and ‘sub-root layer’, respectively. Root dry weight (RDW) was determined by weighing the oven-dried samples after scanning. Specific root length (SRL) was calculated from root length and root dry weight, and root/shoot ratio (RSR) was assessed from RDW and shoot dry weight (SDW), respectively. Root tissue density (RTD) was calculated as root mass/root volume. Root length density (RLD) was calculated as the total root length per unit area. The measured traits were divided into global and local traits depending on the object of description. Global referred to the whole root and shoot, and local referred to the roots at different depths. The detailed description of all assessed traits was shown in Table 1. The relative increase in root length under stress treatment ∆RL was calculated from root length under stress (RL_S_) and control root length (RL_C_) [65]:

(1) $$∆RL=\left({RL}_{S}-{RL}_{C}\right)/{RL}_{C}$$

Plant P concentration was measured by the molybdo-vanadophosphate method after samples were digested with concentrated H_2_SO_4_ and H_2_O_2_ [66]. Plant P uptake was then calculated from plant dry weight and P concentration. Phosphorus acquisition efficiency (PAE) was calculated as follows [67]:

(2) $$PAE={(P}_{S}/P_{C})\times 100\%$$

Here, PAE is phosphorus acquisition efficiency, P_S_ is phosphorus content of plants under stress treatments, and P_C_ is phosphorus content of control plants.

The stress tolerance score (STS) for each genotype was calculated as follows [68,69]:

(3) STSn=SSIn+TIn+SIn+HMIn+MPIn+GMPIn+STIn

(4) $${STS}_{SUM}={STS}_{D}+{STS}_{DLP}+{STS}_{LP}$$

where

Stress sensitivity index $SSI=(1-{DW}_{S}/{DW}_{C})/(1-\bar{{DW}_{S}}/\bar{{DW}_{C}}$),
Tolerance index $TI={DW}_{C}-{DW}_{S}$,
Stress index $SI={DW}_{S}/{DW}_{C}$,
Harmonized mean index $HMI=2({DW}_{C}\times {DW}_{S})/\left({DW}_{C}+{DW}_{S}\right)$,
Mean productivity index $MPI=({DW}_{C}+{DW}_{S})/2$,
Geometric mean productivity index $GMPI=\sqrt{{DW}_{C}\times {DW}_{S}}$,
Stress tolerance index $STI=\left({DW}_{C}\times {DW}_{S}\right)/{(\bar{{DW}_{C})}}^{2}$.

where n is the corresponding genotype number, ${DW}_{C}$ is the dry weight of the control plants, ${DW}_{S}$ is the dry weight of the stress-treated plants, $\bar{{DW}_{C}}\;$ is the average total dry weight of the plants of all the genotypes of the control, $\bar{{DW}_{S}}$ is the average total dry weight of plants of all the genotypes of the stress treatment.

### 4.3. Statistics and Analysis

The data statistical analysis was conducted using the R.4.0.3 software package [70]. A general linear model (GLM) multivariate analysis was performed for genotype main effects after identifying non-significant differences between bins and harvest times using R 4.0.3 software. ANOVA analysis was calculated with Tukey’s HSD (*p* < 0.05). General correlation analysis was carried out by the corrplot package [71] between each pair of traits, and Pearson’s correlation was considered statistically significant at *p* ≤ 0.05. The broad-sense heritability under the four treatments and combined, and the variance due to genotype × treatment interaction was measured as follows [72,73]:

(5) $$H=V_{G}/(V_{G}+V_{\varepsilon }/r)$$

(6) $$H_{com}=V_{G}/(V_{G}+V_{GE}/e+V_{\varepsilon }/re)$$

where V_G_, V_ε_, and V_GE_ are the genotypic variance, error variance, and genotype by treatment variance, respectively. “e” is the number of treatments, and “r” is the replication.

The same set of traits was also utilized for principal component analysis (PCA) of global and local traits to determine the determinants of root morphological variation among genotypes using the FactoMineR package [74] for extracting and visualizing the results. Cluster analysis was performed using the Euclidean distance method by the factoextra package [75]. Figures were created using the ggplot2 package [76]. The 10 characteristic traits of each group genotype in cluster analysis were normalized and displayed in bar chart. Normalization formula [77]:

(7) $$x^{\prime }=\frac{x}{\sum_{i=1}^{n}x_{i}}$$

x′ is the normalized value, x is the average value of the traits in each group, and the denominator is the sum of the average values of all groups.

The natural log-transformed response ratio (LnR) was employed to measure the effect size of each trait under different stress treatments (Equation (7)). Percentage transformations were applied to the effect sizes (Equation (8)), and the variance (v) was calculated for each effect size (Equation (9)) [78]:

(8) $$LnR=\ln\left(V_{s}/V_{c}\right)$$

(9) $$\%∆=100\times (\exp\left(LnR\right)-1)$$

(10) $$v={S_{s}}^{2}/n_{s}{V_{s}}^{2}+{S_{c}}^{2}/n_{c}{V_{c}}^{2}$$

In the above equations, Vs and Vc are the means of the traits under stress treatments and control, respectively. where $n_{s}$ and $n_{c}$ are the sample sizes of the stress treatments and control groups, respectively; Ss and Sc are the standard deviations of the stress and control groups, respectively.

## 5. Conclusions

A significant diversity in root traits was evident across the 100 winter wheat genotypes examined. More than 80% of the assessed traits were notably influenced by low P stress and PEG-induced drought stress, alone or in combination. Winter wheat exhibited adaptations to both stresses, such as decreased biomass and P content alongside increased root length and diameter in deeper layers. When subject to combined stress, wheat plants displayed reduced leaf number, intensified reduction in biomass and P content, and diminished extension of the root system into deeper layers. The augmentation of root length notably contributes to P acquisition during drought and low P stress conditions. The heritability and stress tolerance of root traits in winter wheat are contingent upon the specific stress conditions. Certain highly heritable traits, such as root number and leaf number, exhibit potential as parameters for future breeding programs aimed at enhancing environmental adaptability, resource use efficiency, and grain yield. Drought and low P tolerance genotypes Qinnong 29 and Xinong 979, along with root-adaptive genotype Yunnong 168, could serve as promising candidates for drought-resistant and P-efficient breeding programs. The utilization of a high-throughput phenotype system holds promise in expediting breeding processes, minimizing breeding costs, and when complemented with field trials, facilitating the identification of crop varieties equipped with adaptable root systems. This approach is particularly crucial for future screening and breeding endeavors.

## Figures and Tables

**Table 1:** Description of 14 root-related traits (global traits) and 10 shoot-related traits in 100 wheat genotypes characterized in a semi-hydroponic phenotyping system.

Trait	Abbreviation	Description	Unit
Maximum root depth	MRD	The longest seminal root length	cm
Root number	RN	Seminal and primary root number (from seeds)	Number per plant
Root length	RL	Total root length per plant	cm
Root diameter	RD	Average root diameter	mm
Root area	RA	Total root surface area	cm^2^
Root volume	RV	Total root volume	cm^3^
Root length density	RLD	Root length per unit area (0–110 cm depth, 1430 cm^2^)	cm·cm^−2^
Specific root length	SRL	Total root length per unit root dry mass	mg^−1^ dry mass
Root length Intensity	RLI	Total root length per unit root depth	cm·cm^−1^
Root tissue density	RTD	Root dry mass per unit root volume	mg·cm^−3^
Root growth rate	RGR	Average daily root growth (based on the longest seminal or primary root growth at 65 days after transplanting)	cm·d^−1^
Root dry weight	RDW	Root dry weight per plant	g
Root phosphorus concentration	RPC	Root phosphorus concentration per gram dry weight	mg·g^−1^
Root phosphorus content	RP	Root phosphorus content per plant	mg
Shoot dry weight	SDW	Shoot dry weight per plant	g
Root–shoot ratio	RSR	Root-to-shoot dry mass ratio	
Total dry mass	TDM	Total dry mass (sum of root and shoot dry weight)	mg
Shoot height	SH	Shoot height measured to the tallest leaf	cm
Leaf number	LN	Number of leaves per plant	
Tiller number	TN	Number of tillers per plant	
Shoot growth rate	SGR	Average daily shoot growth	cm·d^−1^
Shoot phosphorus concentration	SPC	Shoot phosphorus concentration per gram of dry weight	mg·g^−1^
Shoot phosphorus content	SP	Shoot phosphorus content per plant	mg
Total phosphorus content	TP	Total phosphorus content per plant	mg

**Table 2:** Descriptive statistics of the coefficient of variation (CV) and mean for 100 genotypes of the 12 measured global traits (CV > 0.3 in control treatment) under four treatments (C, LP, D and DLP).

Trait	CV				Mean			
	C	LP	D	DLP	C	LP	D	DLP
MRD	**0.31**	0.28	**0.36**	0.27	55.10 c	79.87 a	73.29 b	74.81 b
RL	**0.61**	**0.52**	**0.51**	**0.45**	1290.21 b	1390.51 b	1846.70 a	1242.26 b
RD	**0.50**	**0.50**	**0.40**	**0.40**	0.18 b	0.24 a	0.24 a	0.23 a
RA	**0.57**	**0.50**	**0.52**	**0.45**	97.24 b	103.15 b	143.20 a	91.33 b
RV	**0.79**	**0.85**	**0.66**	**0.84**	0.66 b	0.69 b	0.96 a	0.62 b
SRL	**0.73**	**0.64**	**0.95**	**0.48**	12954.18 b	16974.74 a	17172.59 a	18690.97 a
RLI	**0.65**	**0.54**	**0.68**	**0.57**	24.88 b	18.37 c	28.46 a	17.83 c
RTD	**1.12**	**1.59**	**1.62**	**1.09**	0.27 a	0.22 ab	0.26 a	0.18 b
RDW	**0.51**	**0.32**	**0.49**	0.28	0.12 b	0.09 c	0.14 a	0.07 d
SDW	**0.48**	**0.37**	**0.53**	**0.38**	0.52 a	0.12 c	0.16 b	0.11 c
TDM	**0.45**	0.27	**0.42**	0.29	0.63 a	0.20 c	0.30 b	0.18 c
RSR	**0.54**	**0.52**	**1.47**	**0.56**	0.25 c	0.85 b	1.19 a	0.77 b

Trait descriptions and units see Table 1 and Table S1. Coefficient of variation values for traits with CV ≥ 0.3 are shown in bold. Different lowercase letters indicate statistically significant differences (Tukey’s HSD at *p* ≤ 0.05) among treatments. C is control treatment; LP is low phosphorus stress; and D is PEG-induced drought stress; DLP is combined drought and low phosphorus stress. MRD, maximum root depth; RL, root length; RD, root diameter; RA, root area; RV, root volume; SRL, specific root length; RLI, root length intensity; RTD, root tissue density; RDW, root dry weight; SDW, shoot dry weight; TDM, total dry mass; RSR, root–shoot ratio.

**Table 3:** Pearson correlation analysis of stress tolerance score (STS), phosphorus acquisition efficiency (PAE), and the variation of root length and root diameter in relation to the control ones (ΔRL and ΔRD) of 100 genotypes under three stress treatments. * *p* ≤ 0.05, ** *p* ≤ 0.01, ns = not significant.

	Items	LP	D	DLP
STS	PAE	−0.730 **	−0.668 **	−0.617 **
	△RL	−0.456 **	−0.532 **	−0.425 **
	△RL_s1	−0.285 **	−0.452 **	−0.293 **
	△RL_sub	−0.468 **	−0.455 **	−0.426 **
	△RD	−0.217 *	−0.122 ns	−0.071 ns
	△RD_s1	0.088 ns	−0.044 ns	0.026 ns
	△RD_sub	−0.246 *	−0.142 ns	−0.104 ns
PAE	△RL	0.510 **	0.588 **	0.515 **
	△RL_s1	0.373 **	0.495 **	0.431 **
	△RL_sub	0.497 **	0.480 **	0.493 **
	△RD	0.277 *	0.190 ns	0.165 ns
	△RD_s1	−0.083 ns	0.045 ns	0.129 ns
	△RD_sub	0.296 **	0.210 *	0.186 ns

C is control treatment; LP is low phosphorus stress; and D is PEG-induced drought stress; DLP is combined drought and low phosphorus stress. s1 represents the 0–20 cm root segments. Sub represents the root segments below 20 cm.

## Data Availability

The original contributions presented in the study are included in the article/Supplementary Materials, further inquiries can be directed to the corresponding authors.

## Supplementary Materials

The following supporting information can be downloaded at: https://www.mdpi.com/article/10.3390/plants13233361/s1, Figure S1. Correlation of root traits with shoot dry weight. Figure S2. Correlation between root length of different root segments and total root dry weight of plants. Figure S3. Principal component analysis of dry weight-based stress tolerance indexes under three stress conditions. Figure S4. Dendrogram of cluster analysis of 100 winter wheat cultivars based on stress tolerance scores under three stress treatments. Table S1. Description of 26 root-related traits (local traits) in 100 wheat genotypes characterized in a semi-hydroponic phenotyping system. Table S2. Table S2. The F value of three-way analysis of variance of all traits in genotypes (G), phosphorus supply (P), and water status (W). Table S3. Descriptive statistics of the coefficient of variation (CV) and mean for 100 genotypes of the 38 measured traits under four treatments (C, LP, D and DLP). Table S4. Descriptive statistics of the range of maximum and minimum values for 100 genotypes of measurement traits under four treatments. Table S5. Variable loading scores of 12 selected global traits and the proportion of variation of each principal component. Table S6. Ranking of top 10 and bottom 10 of stress tolerance-related indices for 100 genotypes under different treatments. Table S7. Broad-sense heritability estimates for the 23 tested traits under the four different treatments. Table S8. Information on 100 winter wheat genotypes used in this study and descriptions of their stress tolerance scores based on this study.

## Abbreviations

AHC: the clustering hierarchical clustering; CV, the coefficients of variation; DAT, days after transplanting; G, Genotypes; GLM, general linear model; LN, leaf number; MRD, maximum root depth; P, phosphorus; PAE, Phosphorus acquisition efficiency; RA, root area; RA_s1, root area s1; RA_s2, root area s2; RA_s3, root area s3; RA_s4, root area s4; RA_sub, root area in sub-layer; RD, root diameter; RD_s1, root diameter s1; RD_s2, root diameter s2; RD_s3, root diameter s3; RD_s4, root diameter s4; RD_sub, root diameter in sub-layer; RDW, root dry weight; RGR, root growth rate; RL, root length; RL_s1, root length s1; RL_s2, root length s2; RL_s3, root length s3; RL_s4, root length s4; RL_sub, root length in sub-layer; RLD, Root length density; RLD_s1, Root length density s1; RLD_s2, Root length density s2; RLD_s3, Root length density s3; RLD_s4, Root length density s4; RLD_sub, Root length density in sub-root layer; RLI, root length intensity; RLR_s1/sub, Root length ratio; RN, root number; RP, root phosphorus content; RPC, root phosphorus concentration; RSR, root–shoot ratio; RTD, root tissue density; RV, root volume; RV_s1, root volume s1; RV_s2, root volume s2; RV_s3, root volume s3; RV_s4, root volume s4; RV_sub, root volume in sub-layer; SDW, shoot dry weight; SGR, shoot growth rate; SH, shoot height; SP, shoot phosphorus content; SPC, shoot phosphorus concentration; SRL, specific root length; STS, stress tolerance score; TDM, total dry mass; TN, tiller number; TP, total phosphorus content; W, water status.
