# Supplementary material for: Genotypic Variability in Root Morphology in a Diverse Wheat Genotypes Under Drought and Low Phosphorus Stress
Source: Plants (Basel). 2024 Nov 29;13(23):3361. doi: 10.3390/plants13233361 (PMC11644076; doi:10.3390/plants13233361)
Supplement: Supplementary file 1 [file plants-13-03361-s001.zip › supplementary figures.pdf]

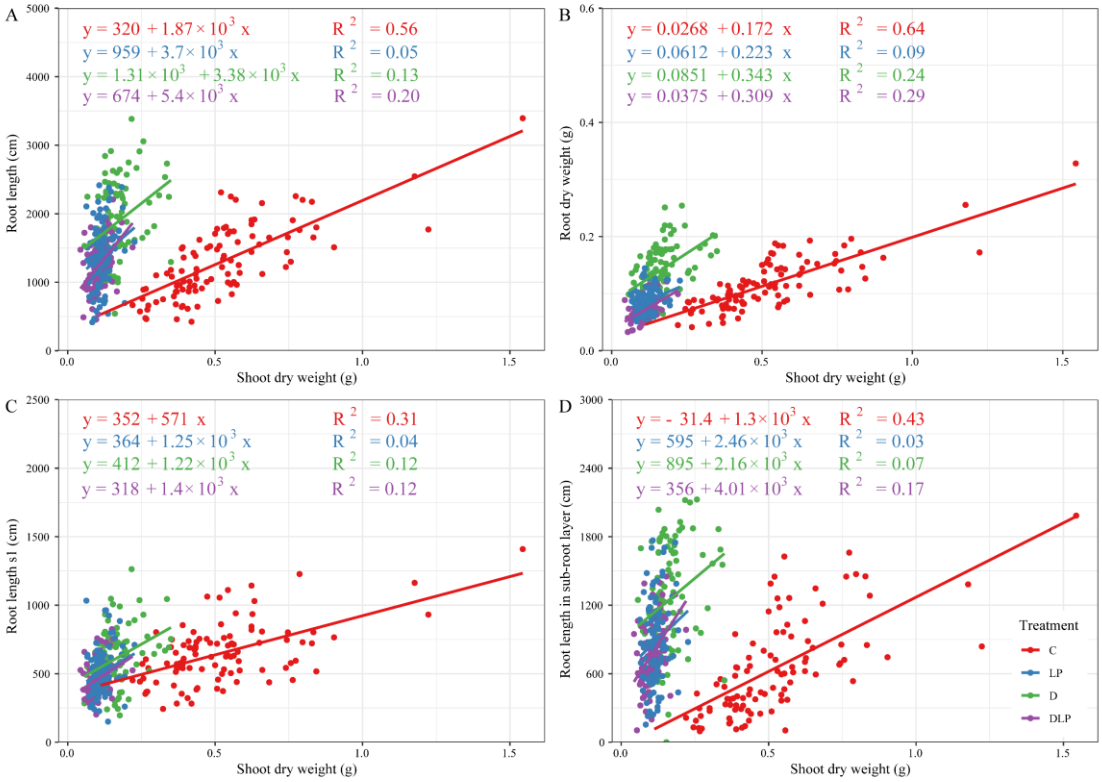

Figure S1. Correlation of root traits with shoot dry weight. A, Scatter plot of root length and shoot dry weight. B, Scatter plot of root dry weight and shoot dry weight. C, Scatter plot of shoot dry weight and root length for s1 (0-20 cm) root segments. D, Scatter plot of shoot dry weight and root length for sub-root segments (below 20 cm). Different colors represent different treatments.

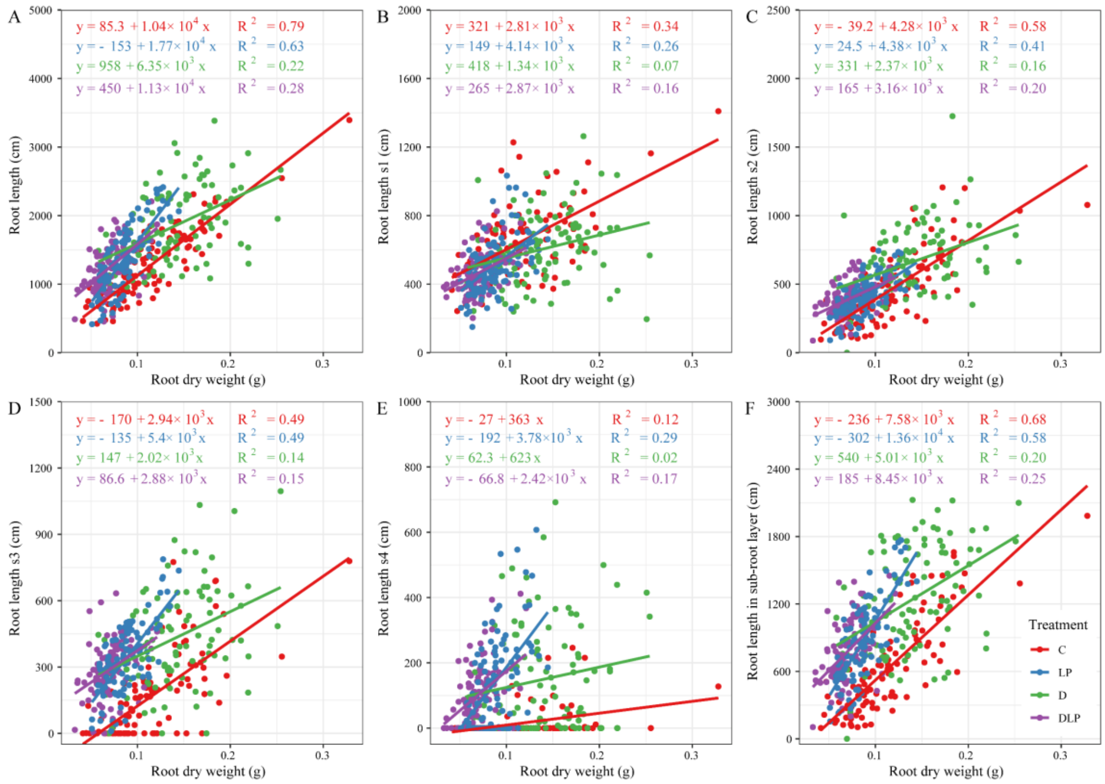

Figure S2. Correlation between root length of different root segments and total root dry weight of plants. A, total root length. B, 0-20 cm (s1) root length. C, 20-40 cm (s2) root length. D, 40-60 cm (s3) root length. E, s4 (below 60 cm) root length. F, below 20 cm (sub) root length, versus total dry weight of the root. Different colors represent different treatments.

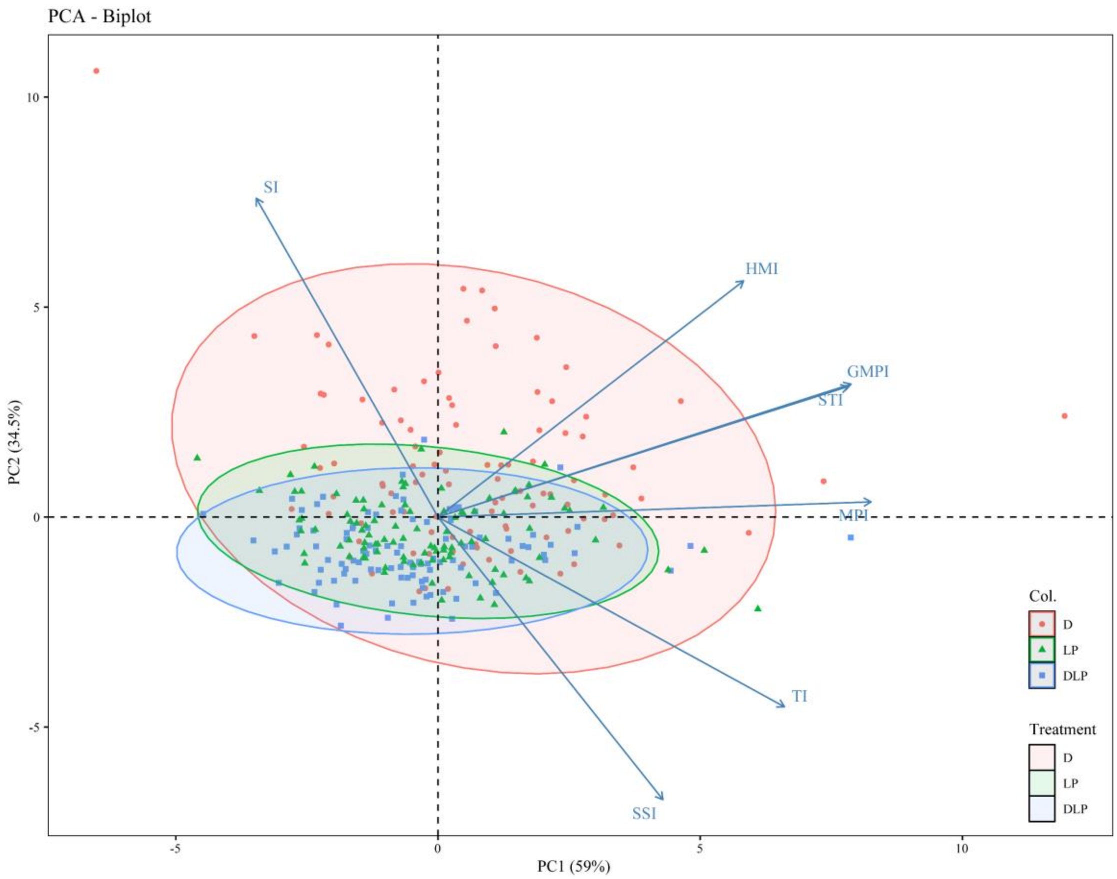

Figure S3. Principal component analysis of dry weight-based stress tolerance indexes under three stress conditions. SSI, Stress sensitivity index; TI, Tolerance index; SI, Stress index; HMI, Harmonized mean index; MPI, Mean productivity index; GMPI, Geometric mean productivity index; STI, Stress tolerance index.

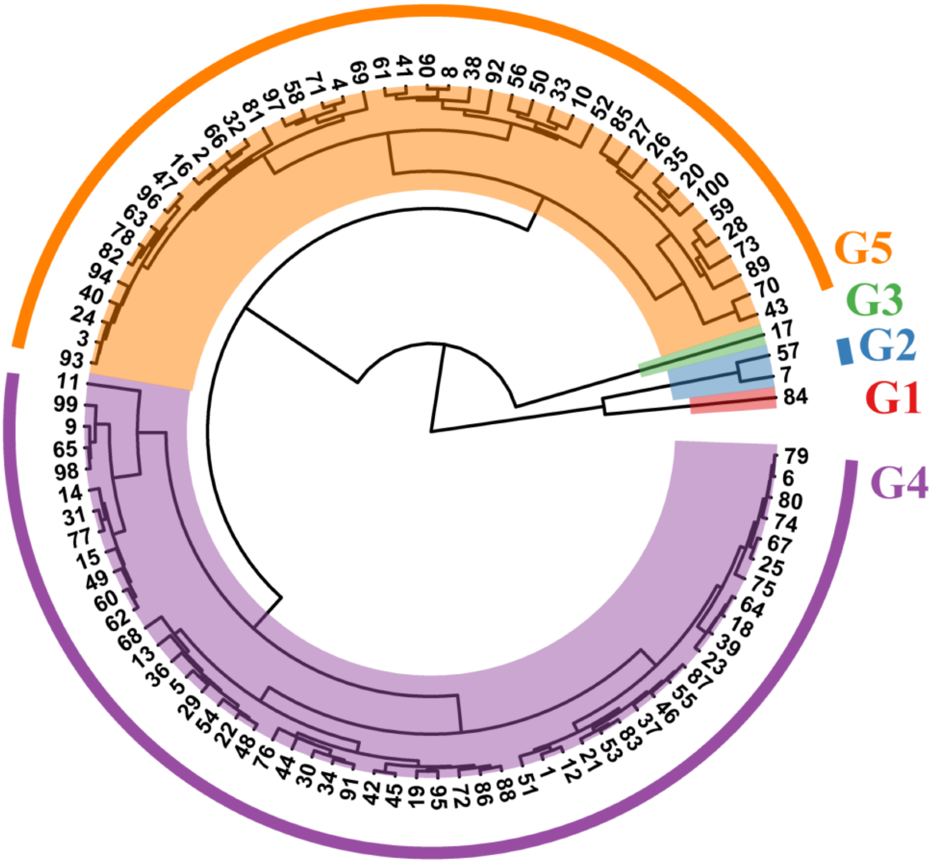

Figure S4. Dendrogram of cluster analysis of 100 winter wheat cultivars based on stress tolerance scores under three stress treatments. Different colors represent different groupings only. 1, Huazhang 166; 2, Fengdecunmai 21; 3, Yongminmai No.1; 4, Pumai 116 ; 5, Yongfeng103; 6, Zhongzhimai No.13; 7, Xinong 979; 8, Fengdeicunmai 22; 9, Hangyu 33; 10, Saidemai No.8; 11, Womai 33; 12, Deyan 0516; 13, Baimai 312; 14, Huaimai 40; 15, Zhengmai 16; 16, Huaimai 226; 17, Xinong 556; 18, Lankao 298; 19, Xinmai 2111; 20, Hangyu 33; 21, Bainong 418; 22, Yongminmai No.1; 23, Zhengmai 518; 24, Saidemai 601; 25, Xinong 0615; 26, Huaimai 920; 27, Zhongliang 91250; 28, Shannong 7064; 29, Hefeng No.3; 30, Shunmai No.11; 31, Minfeng 266; 32, Zhoumai 18; 33, Xuke No.6; 34, Cunmai 633; 35, Bainong 558; 36, Luomai 26; 37, Ruiquanmai 32; 38, Qinnong 168; 39, Tainong 33; 40, Lunxuan 2000; 41, Lankaoai 6; 42, Yikemai No.5; 43, Huaihe 12148; 44, Bonong No.6; 45, Xinmai 21; 46, Yanzhan 4110; 47, Xunong No.10; 48, Tunmai 257; 49, Xinong 501; 50, Longpingmai No.3; 51, Cunmai 30; 52, Zhongmai 166; 53, Huaimai 1403; 54, Fumai 0808; 55, Xinong 20; 56, Jimai 44; 57, Yunong 168; 58, Baoliang No.5; 59, Saidemai 601; 60, Suyu 0622; 61, Xiaoyan 22; 62, Baofeng 1530; 63, Shannong 116; 64, Pingan 0658; 65, Qinnong 578; 66, Quanmai31; 67, Saidemai No.2; 68, Xinong 235; 69, Qinnong 28-6; 70, Zhongyu 1401; 71, Gaoke 115; 72, Luomai 906; 73, Huacheng 5157; 74, Bainong 207; 75, Huaihe 13068; 76, Taihemai No.6; 77, Fengdecunmai 16; 78, Xinzhi No.6; 79, Shunmai No.10; 80, Wunong 68; 81, Xunong 029; 82, Xuke 718; 83, Jinxiu 21; 84, Qinnong 29; 85, Yumai 64; 86, Xinmai 38; 87,

Qinmai 618; 88, Longke 1221; 89, Bainong 419; 90, Xiansheng 368; 91, Zhongyuan 20; 92, Huamai 304; 93, Tianmai 186; 94, Zhongmai 99; 95, Zhongzhongmai 27; 96, Yanfeng 168; 97, Huaimai 1196; 98, Zhongzhongmai 18; 99, Zhongzhimai No.13; 100, Zhengmai 9023.
