# Supplementary material for: Genotypic Variability in Root Morphology in a Diverse Wheat Genotypes Under Drought and Low Phosphorus Stress
Source: Plants (Basel). 2024 Nov 29;13(23):3361. doi: 10.3390/plants13233361 (PMC11644076; doi:10.3390/plants13233361)
Supplement: Supplementary file 1 [file plants-13-03361-s001.zip › supplementary tables.pdf]

Table S1. Description of 26 root-related traits (local traits) in 100 wheat genotypes characterized in a semi-hydroponic phenotyping system.

Trait	Abbreviation	Description	Unit
**Root length s1**	RL_s1	Total root length in 0–20 cm (s1, top-root layer)	cm
**Root diameter s1**	RD_s1	Average root diameter in 0–20 cm (s1)	mm
**Root area s1**	RA_s1	Total root surface area in 0–20 cm (s1)	cm^2
**Root volume s1**	RV_s1	Total root volume in 0–20 cm (s1)	cm^3
**Root length density s1**	RLD_s1	Root length per unit area in 0–20 cm (s1)	$cm{\cdot}cm^{-2}$
**Root length s2**	RL_s2	Total root length in 20–40 cm (s2)	cm
**Root diameter s2**	RD_s2	Average root diameter in 20–40 cm (s2)	mm
**Root area s2**	RA_s2	Total root surface area in 20–40 cm (s2)	cm^2
**Root volume s2**	RV_s2	Total root volume in 20–40 cm (s2)	cm^3
**Root length density s2**	RLD_s2	Root length per unit area in 20–40 cm (s2, 260 cm^2)	$cm{\cdot}cm^{-2}$
**Root length s3**	RL_s3	Total root length in 40–60 cm (s3)	cm
**Root diameter s3**	RD_s3	Average root diameter in 40–60 cm (s3)	mm
**Root area s3**	RA_s3	Total root surface area in 40–60 cm (s3)	cm^2
**Root volume s3**	RV_s3	Total root volume in 40–60 cm (s3)	cm^3
**Root length density s3**	RLD_s3	Root length per unit area in 40–60 cm (s3, 260 cm^2)	$cm{\cdot}cm^{-2}$
**Root length s4**	RL_s4	Total root length in below 60 cm (s4)	cm
**Root diameter s4**	RD_s4	Average root diameter in below 60 cm (s4)	mm
**Root area s4**	RA_s4	Total root surface area in below 60 cm (s4)	cm^2
**Root volume s4**	RV_s4	Total root volume in below 60 cm (s4)	cm^3
**Root length density s4**	RLD_s4	Root length per unit area in below 60 cm (s4, 650 cm^2)	$cm{\cdot}cm^{-2}$
**Root length in sub-root layer**	RL_sub	Combined root length in below 20 cm (sub-root layer)	cm
**Root diameter in sub-root layer**	RD_sub	Combined average root diameter in below 20 cm (sub-root layer)	mm
**Root area in sub-root layer**	RA_sub	Total root surface area in below 20 cm (sub-root layer)	cm^2
**Root volume in sub-root layer**	RV_sub	Total root volume in below 20 cm (sub-root layer)	cm^3
**Root length density in sub-root layer**	RLD_sub	Root length per unit area in below 20 cm (sub-root layer)	$cm{\cdot}cm^{-2}$
**Root length ratio**	RLR_s1/sub	Root length in s1 over sub-root layer	

Table S2. The F value of three-way analysis of variance of all traits in genotypes (G), phosphorus supply (P), and water status (W). $^{*}p \leq 0.05$, $^{**}p \leq 0.01$, $^{***}p \leq 0.001$, **ns= not significant.**

Trait	G	P	W	G*P	G*W	P*W	G*P*W
**MRD**	1.950***	115.42***	28.79***	0.85ns	0.86ns	90.35***	0.95ns
**RN**	11.13***	609.42***	3676.79***	8.83***	6.89***	1971.18***	7.62***
**RL**	2.11***	36.33***	23.82***	1.07ns	0.84ns	71.00***	1.37*
**RD**	5.60***	28.93***	34.25***	1.16ns	1.18ns	49.01***	0.95ns
**RA**	2.35***	55.47***	30.58***	1.17ns	0.92ns	87.67***	1.26ns
**RV**	5.34***	27.71***	16.35***	0.92ns	0.82ns	41.62***	0.70ns
**RLD**	2.11***	36.33***	23.82***	1.07ns	0.84ns	71.00***	1.37*
**SRL**	0.85ns	14.72***	16.90***	0.75ns	0.55ns	3.00ns	0.49ns
**RLI**	1.02ns	98.27***	3.08ns	0.67ns	0.67ns	5.68*	0.88ns
**RTD**	1.81***	9.87**	1.50ns	0.39ns	0.83ns	0.56ns	0.61ns
**RGR**	1.950***	115.42***	28.79***	0.85ns	0.86ns	90.35***	0.95ns
**RDW**	1.71***	406.04***	1.96ns	1.50**	1.93***	70.91***	2.56***

**RPC**	2.02***	99.17***	167.99***	2.03***	1.59***	41.03***	1.94***
**RP**	1.01ns	2141.05***	859.52***	0.99ns	1.29*	40.47***	0.89ns
**SDW**	4.86***	1888.88***	1241.72***	4.39***	4.36***	1091.55***	4.96***
**TDM**	5.28***	2141.89***	917.06***	4.49***	5.11***	649.79***	5.97***
**RSR**	1.14ns	2.84ns	66.79***	1.22ns	1.09ns	96.06***	1.20ns
**SH**	3.70***	1012.61***	239.65***	1.14ns	0.72ns	305.11***	1.26ns
**LN**	9.24***	4249.70***	377.31***	7.35***	6.13***	338.08***	7.13***
**TN**	9.09***	100.00***	100.00***	9.09***	9.09***	100.00***	9.09***
**SGR**	3.70***	1012.61***	239.65***	1.14ns	0.72ns	305.11***	1.26ns
**SPC**	1.28*	4554.70***	140.12***	1.33*	1.27*	11.30***	1.18ns
**SP**	4.89***	2547.27***	1314.69***	4.66***	4.62***	1155.97***	5.06***
**TP**	4.88***	2946.95***	1495.17***	4.63***	4.57***	1188.19***	5.05***
**RL_s1**	2.11***	73.63***	6.95**	1.03ns	0.92ns	0.00ns	0.92ns
**RD_s1**	2.44***	11.64***	2.63ns	0.62ns	0.64ns	0.28ns	0.86ns
**RA_s1**	1.84***	114.18***	6.88**	0.99ns	0.94ns	0.05ns	0.89ns
**RV_s1**	2.40***	93.42***	5.40*	0.80ns	0.84ns	0.13ns	0.82ns
**RLD_s1**	2.11***	73.63***	6.95**	1.03ns	0.92ns	0.00ns	0.92ns
**RL_s2**	1.68***	77.22***	24.70***	0.75ns	1.06ns	36.74***	1.11ns
**RD_s2**	3.45***	0.90ns	1.35ns	0.80ns	0.40ns	2.16ns	0.96ns
**RA_s2**	1.59***	87.27***	29.33***	0.79ns	0.90ns	42.55***	0.90ns
**RV_s2**	3.57***	37.82***	12.82***	0.72ns	0.58ns	11.89***	0.87ns
**RLD_s2**	1.68***	77.22***	24.70***	0.75ns	1.06ns	36.74***	1.11ns
**RL_s3**	1.82***	0.76ns	54.69***	1.57***	1.09ns	116.63***	1.67***
**RD_s3**	3.10***	17.65***	25.76***	1.29*	1.69***	34.56***	1.06ns
**RA_s3**	2.10***	0.49ns	71.16***	1.67***	1.33*	145.78***	1.70***
**RV_s3**	3.15***	0.85ns	23.10***	0.94ns	1.23ns	50.25***	0.91ns
**RLD_s3**	1.82***	0.76ns	54.69***	1.57***	1.09ns	116.63***	1.67***
**RL_s4**	4.00***	36.87***	62.35***	3.22***	3.10***	184.74***	4.46***
**RD_s4**	5.87***	103.34***	89.15***	2.68***	3.10***	104.12***	2.76***
**RA_s4**	4.91***	29.66***	70.78***	3.23***	3.39***	189.36***	4.09***
**RV_s4**	4.74***	9.20**	30.62***	1.70***	2.29***	54.97***	1.62***
**RLD_s4**	4.00***	36.87***	62.35***	3.22***	3.10***	184.74***	4.46***
**RL_sub**	2.43***	14.03***	67.31***	1.36*	1.04ns	136.22***	1.88***
**RD_sub**	5.57***	49.35***	49.88***	1.42**	1.38*	60.25***	1.19ns
**RA_sub**	2.97***	19.39***	90.52***	1.60***	1.18ns	178.15***	1.86***
**RV_sub**	6.03***	5.15*	42.03***	1.24ns	0.96ns	69.99***	0.96ns
**RLD_sub**	2.43***	14.03***	67.31***	1.36*	1.04ns	136.22***	1.88***
**RLR_s1/sub**	3.16***	55.21***	109.18***	1.62***	1.58***	144.66***	2.05***

MRD, maximum root depth; RN, root number; RL, root length; RD, root diameter; RA, root area; RV, root volume; RLD, Root length density; SRL, specific root length; RLI, root length intensity; RTD, root tissue density; RGR, root growth rate; RDW, root dry weight; RPC, root phosphorus concentration; RP, root phosphorus content; SDW, shoot dry weight; TDM, total dry mass; RSR, root-shoot ratio; SH, shoot height; LN, leaf number; TN, tiller number; SGR, shoot growth rate; SPC, shoot phosphorus concentration; SP, shoot phosphorus content; TP, total phosphorus content; RL_s1, root length s1; RD_s1, root diameter s1; RA_s1, root area s1; RV_s1, root volume s1; RLD_s1, Root length density s1; RL_s2, root length s2; RD_s2, root diameter s2; RA_s2, root area s2; RV_s2, root volume s2; RLD_s2, Root length density s2; RL_s3, root length s3; RD_s3, root diameter s3; RA_s3, root area s3; RV_s3, root volume s3; RLD_s3, Root length density s3; RL_s4, root length s4; RD_s4, root diameter s4; RA_s4, root area s4; RV_s4, root volume s4; RLD_s4, Root length density s4; RL_sub, root length in sub layer; RD_sub, root diameter in sub layer; RA_sub, root area in sub layer; RV_sub, root volume in sub layer; RLD_sub, Root length density in sub-root layer; RLR_s1/sub, Root length ratio. Trait descriptions and units see Table 1.

Table S3. Descriptive statistics of the coefficient of variation (CV) and mean for 100 genotypes of the 38 measured traits under four treatments (C, LP, D and DLP).

Trait	CV				mean			
	C	LP	D	DLP	C	LP	D	DLP
RN	0.18	0.16	0.17	0.19	8.64 a	6.19b	4.91 d	5.61 c
RLD	**0.61**	**0.52**	**0.51**	**0.45**	0.94 b	1.01 b	1.34 a	0.90 b
RGR	**0.31**	0.28	**0.36**	0.27	0.85 c	1.23 a	1.13 b	1.56 b
RPC	**0.51**	**0.35**	**0.39**	**0.35**	1.67 a	1.05 b	0.94 bc	0.81 c
RP	0.07	0.16	0.14	0.25	0.15 a	0.08 c	0.10 b	0.05 d
SH	0.12	0.14	0.17	0.17	43.10 a	30.00 c	34.34 b	30.53 c
LN	0.15	0.12	0.12	0.12	8.07 a	5.66 c	6.98 b	5.63 c
TN	**0.30**	0.00	0.00	0.00	1.10 a	1.00 b	1.00 b	1.00 b
SGR	0.12	0.14	0.17	0.17	0.66 a	0.46 c	0.53 b	0.47 c
SPC	0.07	0.07	0.08	0.10	2.76 a	1.69 c	2.53 b	1.56 d
SP	**0.43**	0.27	**0.41**	**0.31**	1.44 a	0.20 c	0.41 b	0.16 c
TP	**0.39**	0.20	**0.33**	0.25	1.59 a	0.28 c	0.51 b	0.22 c
RL_s1	**0.51**	**0.50**	**0.59**	**0.42**	647.56 a	509.31 b	605.83 a	465.41 b
RD_s1	**0.31**	**0.47**	0.25	0.27	0.26 a	0.25 ab	0.26 a	0.24 b
RA_s1	**0.49**	**0.48**	**0.62**	**0.43**	50.75 a	37.50 b	47.71 a	33.88 b
RV_s1	**0.61**	**0.75**	**0.73**	**0.65**	0.33 a	0.24 ab	0.31 a	0.21 b
RLD_s1	**0.51**	**0.50**	**0.59**	**0.42**	2.59 a	2.04 b	2.42 a	1.86 b
RL_s2	**0.88**	**0.63**	**0.60**	**0.57**	457.26 b	406.64 b	662.05 a	386.37 c
RD_s2	**0.62**	**0.50**	**0.38**	**0.63**	0.26 a	0.24 b	0.24 a	0.24 a
RA_s2	**0.80**	**0.59**	**0.59**	**0.57**	31.97 b	28.41 bc	47.02 a	27.02 c
RV_s2	**1.01**	**0.95**	**0.67**	**1.19**	0.21 b	0.18 b	0.28 a	0.18 b
RLD_s2	**0.88**	**0.63**	**0.60**	**0.57**	1.83 b	1.63 bc	2.65 a	1.55 c
RL_s3	**1.52**	**0.76**	**0.81**	**0.64**	170.28 c	336.51 b	429.37 a	288.04 b
RD_s3	**1.35**	**0.71**	**0.67**	**0.67**	0.16 b	0.26 a	0.27 a	0.25 a
RA_s3	**1.43**	**0.71**	**0.80**	**0.63**	13.20 c	25.08 b	34.63 a	21.28 b
RV_s3	**2.06**	**1.19**	**0.93**	**1.34**	0.11 c	0.18 b	0.24 a	0.15 bc
RLD_s3	**1.52**	**0.76**	**0.81**	**0.64**	0.68 c	1.35 b	1.72 a	1.15 b
RL_s4	**4.20**	**1.22**	**1.38**	**1.15**	15.12 c	138.05 a	149.45 a	102.44 b
RD_s4	**3.42**	**1.12**	**1.32**	**1.07**	0.02 b	0.20 a	0.19 a	0.19 a
RA_s4	**4.44**	**1.19**	**1.42**	**1.21**	1.33 c	12.16 a	13.83 a	9.14 b
RV_s4	**5.66**	**1.73**	**2.02**	**2.43**	0.01 b	0.10 a	0.12 a	0.08 a
RLD_s4	**4.20**	**1.22**	**1.38**	**1.15**	0.02 c	0.22 a	0.24 a	0.16 b
RL_sub	**0.91**	**0.62**	**0.57**	**0.56**	642.65 c	881.2 b	1240.87 a	776.85 b
RD_sub	**0.71**	**0.59**	**0.52**	**0.50**	0.15 b	0.23 a	0.23 a	0.23 a
RA_sub	**0.84**	**0.61**	**0.58**	**0.55**	46.49 c	65.66 b	95.48 a	57.44 b
RV_sub	**1.18**	**1.03**	**0.75**	**1.09**	0.32 c	0.45 b	0.64 a	0.41 bc
RLD_sub	**0.91**	**0.62**	**0.57**	**0.56**	0.57 c	0.78 b	1.10 a	0.69 b
RLR_s1/sub	**1.06**	**0.67**	**0.78**	**1.34**	1.96 a	0.77 bc	0.58 c	0.86 b

Trait descriptions and units see Table 1 and Table S1. Coefficient of variation values for traits with CV ≥ 0.3 are shown in bold. Different lowercase letters indicate statistically significant differences (Tukey's HSD at $p \leq 0.05$) among treatments. C is control treatment; LP is low phosphorus stress; and D is PEG-induced drought stress; DLP is combined drought and low phosphorus stress. RN, root number; RLD, Root length density; RGR, root growth rate; RPC, root phosphorus concentration; RP, root phosphorus content; SH, shoot height; LN, leaf number; TN, tiller number; SGR, shoot growth rate; SPC, shoot phosphorus concentration; SP, shoot phosphorus content; TP, total phosphorus content; RL_s1, root length s1; RD_s1, root diameter s1; RA_s1, root area s1; RV_s1, root volume s1; RLD_s1, Root length density s1; RL_s2, root length s2; RD_s2, root diameter s2; RA_s2, root area s2; RV_s2, root

volume s2; RLD_s2, Root length density s2; RL_s3, root length s3; RD_s3, root diameter s3; RA_s3, root area s3; RV_s3, root volume s3; RLD_s3, Root length density s3; RL_s4, root length s4; RD_s4, root diameter s4; RA_s4, root area s4; RV_s4, root volume s4; RLD_s4, Root length density s4; RL_sub, root length in sub layer; RD_sub, root diameter in sub layer; RA_sub, root area in sub layer; RV_sub, root volume in sub layer; RLD_sub, Root length density in sub-root layer; RLR_s1/sub, Root length ratio.

Table S4. Descriptive statistics of the range of maximum (max) and minimum (min) values for 100 genotypes of measurement traits under four treatments.

Trait	C		LP		D		DLP	
	min	max	min	max	min	max	min	max
MRD	28.10	88.27	57.22	120.97	43.20	108.20	43.33	108.23
SRN	4.67	13.00	3.67	8.00	4.00	7.00	3.33	9.33
RL	423.48	3393.53	416.15	2414.59	542.13	3383.59	455.99	2206.98
RD	0.10	0.48	0.12	0.56	0.06	0.57	0.11	0.57
RA	31.19	215.58	37.06	183.66	43.84	296.76	30.12	151.36
RV	0.17	2.62	0.24	2.93	0.29	3.20	0.15	3.04
RLD	0.31	2.47	0.30	1.76	0.39	2.46	0.33	1.61
SRL	5835.38	35605.45	7768.17	45004.89	7214.85	59714.35	7021.89	32788.6
RLI	8.68	61.08	5.15	32.68	9.43	57.47	5.19	40.81
RTD	0.05	1.04	0.04	1.44	0.06	1.49	0.02	0.70
RGR	0.43	1.36	0.88	1.86	0.66	1.66	0.67	1.67
RDW	0.04	0.33	0.05	0.14	0.06	0.25	0.03	0.12
RPC	0.43	5.58	0.44	2.66	0.33	2.54	0.34	1.62
RP	0.12	0.17	0.05	0.11	0.07	0.13	0.02	0.08
SDW	0.11	1.54	0.06	0.23	0.06	0.35	0.04	0.22
TDM	0.18	1.87	0.13	0.35	0.16	0.54	0.09	0.32
RSR	0.14	0.63	0.40	1.72	0.39	9.08	0.44	2.70
SH	35.63	56.47	24.23	38.83	27.67	48.20	23.57	48.60
LN	6.00	12.33	4.33	7.00	3.67	8.00	4.00	7.00
TN	1.00	3.00	1.00	1.00	1.00	1.00	1.00	1.00
SGR	0.55	0.87	0.37	0.6	0.43	0.74	0.36	0.75
SPC	2.31	3.18	1.45	2.01	2.02	3.15	1.20	1.88
SP	0.29	4.21	0.10	0.39	0.13	0.95	0.06	0.33
TP	0.44	4.36	0.18	0.47	0.25	1.04	0.09	0.37
RL_s1	243.10	1409.22	150.32	1033.31	195.89	1263.21	202.34	825.47
RD_s1	0.20	0.58	0.19	0.76	0.20	0.40	0.18	0.45
RA_s1	19.31	99.22	12.03	70.45	16.14	91.54	16.52	52.69
RV_s1	0.13	1.03	0.08	0.66	0.09	0.69	0.10	0.62
RLDs1	0.97	5.64	0.60	4.13	0.78	5.05	0.81	3.30
RL_s2	95.90	1205.96	91.27	743.31	0.00	1725.09	88.17	664.61
RD_s2	0.16	0.78	0.17	0.52	0.00	0.53	0.17	1.00
RA_s2	5.36	77.19	6.15	53.67	0.00	89.93	5.09	54.29
RV_s2	0.02	0.97	0.03	0.62	0.00	0.60	0.02	1.36
RLDs2	0.38	4.82	0.37	2.97	0.00	6.90	0.35	2.66
RL_s3	0.00	778.99	10.38	787.29	0.00	1095.29	0.00	633.32
RD_s3	0.00	0.73	0.08	0.86	0.00	1.28	0.00	0.84
RA_s3	0.00	56.12	0.54	56.73	0.00	91.25	0.00	41.53
RV_s3	0.00	0.88	0.00	1.16	0.00	0.84	0.00	0.90
RLDs3	0.00	3.12	0.04	3.15	0.00	4.38	0.00	2.53
RL_s4	0.00	246.04	0.00	607.55	0.00	691.89	0.00	411.65

RD_s4	0.00	0.35	0.00	0.70	0.00	1.24	0.00	0.80
RA_s4	0.00	30.25	0.00	50.12	0.00	76.69	0.00	35.56
RV_s4	0.00	0.35	0.00	0.58	0.00	1.29	0.00	1.06
RLDs4	0.00	0.39	0.00	0.97	0.00	1.11	0.00	0.66
RL_sub	95.90	1984.31	154.39	1767.80	0.00	2125.89	104.22	1451.24
RD_sub	0.06	0.45	0.08	0.61	0.00	0.66	0.08	0.67
RA_sub	5.36	120.90	9.43	133.56	0.00	205.22	6.03	103.38
RV_sub	0.02	1.58	0.05	2.36	0.00	2.52	0.03	2.42
RLD_sub	0.09	1.76	0.14	1.57	0.00	1.89	0.09	1.29
RLR_s1/sub	0.28	8.14	0.28	2.11	0.00	1.97	0.29	5.89

Trait descriptions and units see Table 2. C is control treatment; LP is low phosphorus stress; and D is PEG-induced drought stress; DLP is combined drought and low phosphorus stress. MRD, maximum root depth; RN, root number; RL, root length; RD, root diameter; RA, root area; RV, root volume; RLD, Root length density; SRL, specific root length; RLI, root length intensity; RTD, root tissue density; RGR, root growth rate; RDW, root dry weight; RPC, root phosphorus concentration; RP, root phosphorus content; SDW, shoot dry weight; TDM, total dry mass; RSR, root-shoot ratio; SH, shoot height; LN, leaf number; TN, tiller number; SGR, shoot growth rate; SPC, shoot phosphorus concentration; SP, shoot phosphorus content; TP, total phosphorus content; RL_s1, root length s1; RD_s1, root diameter s1; RA_s1, root area s1; RV_s1, root volume s1; RLD_s1, Root length density s1; RL_s2, root length s2; RD_s2, root diameter s2; RA_s2, root area s2; RV_s2, root volume s2; RLD_s2, Root length density s2; RL_s3, root length s3; RD_s3, root diameter s3; RA_s3, root area s3; RV_s3, root volume s3; RLD_s3, Root length density s3; RL_s4, root length s4; RD_s4, root diameter s4; RA_s4, root area s4; RV_s4, root volume s4; RLD_s4, Root length density s4; RL_sub, root length in sub layer; RD_sub, root diameter in sub layer; RA_sub, root area in sub layer; RV_sub, root volume in sub layer; RLD_sub, Root length density in sub-root layer; RLR_s1/sub, Root length ratio.

Table S5. Variable loading scores of 12 selected global traits and the proportion of variation of each principal component.

Traits	PC1	PC2	PC3	PC4
**MRD**	0.228	**-0.630**	-0.002	0.327
**RL**	**0.905**	-0.082	0.232	-0.122
**RD**	0.430	**-0.602**	-0.490	0.278
**RA**	**0.966**	-0.178	0.016	-0.012
**RV**	**0.747**	-0.301	-0.406	0.170
**SRL**	0.237	-0.456	0.128	**-0.675**
**RLI**	**0.749**	0.300	0.290	-0.364
**RTD**	-0.177	0.354	**0.500**	0.454
**RSR**	0.234	-0.452	**0.573**	0.256
**RDW**	**0.782**	0.402	0.225	0.293
**SDW**	0.301	**0.855**	-0.291	0.002
**TDM**	0.418	**0.843**	-0.219	0.057
**Eigenvalue**	4.1	3.1	1.3	1.2
**Variance (%)**	34.4	26.0	11.0	9.8
**Cumulative variability (%)**	34.4	60.4	71.4	81.2

Twelve global traits with CVs ≥ 0.3 (Table 3) in the control (Excludes RGR and RLD, which are calculated directly from MRD and RL) were used for factor analysis using the principal component analysis (PCA) extraction method. For each trait, the largest variable loading score crossing the two components appears in bold. Principal components with eigenvalues > 1 are presented and considered significant. Trait descriptions and units see Table 2. MRD, maximum root depth; RL, root length; RD, root diameter; RA, root area; RV, root volume; SRL, specific root length; RLI, root length intensity; RTD, root tissue density; RSR, root-shoot ratio; RDW, root dry weight; SDW, shoot dry weight; TDM, total dry mass.

Table S6. Ranking of top 10 and bottom 10 of stress tolerance-related indices for 100 genotypes under different treatments.

Treatment	S.No.	G	SSI	MPI	GMPI	HMI	STI	TI	SI	STS
**LP**	1	#84	1.35	1.01	0.54	0.28	0.72	1.72	0.08	5.70
	2	#7	1.26	0.82	0.55	0.37	0.75	1.22	0.15	5.12
	3	#57	1.28	0.79	0.51	0.33	0.65	1.21	0.13	4.9
	4	#70	1.17	0.64	0.48	0.36	0.58	0.85	0.20	4.29
	5	#43	1.09	0.63	0.51	0.41	0.64	0.73	0.26	4.27
	6	#35	1.07	0.58	0.48	0.39	0.57	0.66	0.28	4.03
	7	#20	1.05	0.57	0.48	0.40	0.57	0.64	0.28	3.99
	8	#100	1.18	0.57	0.43	0.32	0.46	0.77	0.20	3.92
	9	#85	1.23	0.58	0.4	0.28	0.41	0.84	0.16	3.91
	10	#26	1.22	0.57	0.4	0.29	0.41	0.81	0.17	3.88
**D**	1	#84	1.50	1.13	0.86	0.65	1.85	1.48	0.21	7.68
	2	#7	1.47	0.88	0.68	0.52	1.14	1.11	0.22	6.02
	3	#57	1.55	0.83	0.60	0.43	0.89	1.14	0.18	5.61
	4	#20	0.99	0.66	0.62	0.58	0.95	0.47	0.48	4.74
	5	#85	1.34	0.65	0.54	0.45	0.73	0.71	0.29	4.71
	6	#70	1.48	0.65	0.50	0.38	0.62	0.83	0.22	4.68
	7	#35	1.21	0.62	0.55	0.49	0.76	0.58	0.36	4.57
	8	#27	1.37	0.62	0.51	0.42	0.65	0.70	0.28	4.55
	9	#100	1.37	0.61	0.50	0.41	0.63	0.69	0.28	4.50
	10	#59	1.28	0.60	0.52	0.44	0.66	0.61	0.33	4.44
**DLP**	1	#84	1.21	1.05	0.65	0.40	1.06	1.64	0.12	6.14
	2	#57	1.17	0.80	0.54	0.37	0.73	1.18	0.15	4.95
	3	#7	1.21	0.81	0.51	0.32	0.65	1.25	0.13	4.88
	4	#70	1.13	0.63	0.46	0.33	0.52	0.87	0.18	4.12
	5	#43	1.06	0.61	0.48	0.37	0.56	0.77	0.23	4.08
	6	#27	1.09	0.59	0.44	0.34	0.49	0.77	0.21	3.93
	7	#85	1.13	0.59	0.43	0.31	0.46	0.82	0.19	3.92
	8	#26	1.11	0.59	0.43	0.32	0.47	0.79	0.20	3.90
	9	#89	0.86	0.56	0.50	0.44	0.61	0.50	0.38	3.84
	10	#28	1.05	0.55	0.44	0.35	0.48	0.68	0.24	3.78
**LP**	91	#9	0.86	0.26	0.23	0.21	0.14	0.21	0.41	2.33
	92	#31	0.41	0.29	0.29	0.29	0.21	0.09	0.72	2.31
	93	#14	0.74	0.26	0.24	0.23	0.15	0.17	0.50	2.28
	94	#77	0.67	0.26	0.25	0.24	0.16	0.15	0.55	2.28
	95	#62	0.67	0.26	0.25	0.24	0.15	0.15	0.54	2.26
	96	#15	0.52	0.27	0.26	0.26	0.17	0.12	0.65	2.25
	97	#60	0.50	0.26	0.26	0.25	0.17	0.11	0.66	2.21
	98	#49	0.39	0.27	0.26	0.26	0.17	0.08	0.73	2.17
	99	#11	0.41	0.23	0.23	0.22	0.13	0.07	0.73	2.01
	100	#17	0.03	0.18	0.18	0.18	0.08	0.00	0.98	1.64
**D**	91	#9	0.51	0.31	0.31	0.31	0.24	0.10	0.73	2.51
	92	#14	0.50	0.30	0.29	0.29	0.22	0.09	0.74	2.43
	93	#49	0.69	0.25	0.25	0.24	0.15	0.11	0.64	2.33
	94	#77	0.02	0.34	0.34	0.34	0.28	0.00	0.99	2.31

	95	#62	0.00	0.33	0.33	0.33	0.28	0.00	1.00	2.29
	96	#60	0.31	0.29	0.29	0.29	0.21	0.05	0.84	2.28
	97	#31	-0.30	0.37	0.37	0.37	0.34	-0.05	1.16	2.24
	98	#15	-0.41	0.36	0.36	0.36	0.33	-0.07	1.22	2.15
	99	#11	-0.62	0.31	0.31	0.30	0.23	-0.09	1.33	1.77
	100	#17	-3.12	0.34	0.30	0.27	0.22	-0.30	2.64	0.35
**DLP**	91	#65	0.73	0.27	0.25	0.24	0.16	0.20	0.47	2.32
	92	#14	0.69	0.26	0.24	0.23	0.15	0.17	0.50	2.24
	93	#77	0.60	0.27	0.26	0.25	0.16	0.15	0.57	2.24
	94	#31	0.72	0.25	0.24	0.22	0.14	0.18	0.48	2.23
	95	#15	0.76	0.24	0.22	0.20	0.12	0.18	0.45	2.18
	96	#60	0.51	0.26	0.25	0.25	0.16	0.12	0.63	2.18
	97	#62	0.93	0.22	0.19	0.17	0.09	0.22	0.33	2.15
	98	#49	0.76	0.22	0.21	0.19	0.11	0.17	0.45	2.11
	99	#11	0.65	0.20	0.19	0.18	0.09	0.13	0.53	1.98
	100	#17	0.38	0.16	0.16	0.16	0.06	0.05	0.73	1.69

S.No., serial number; G, genotype; SSI, stress susceptibility index; MPI, mean productivity index; GMPI, geometric mean productivity index; HMI, harmonic mean index; STI, stress tolerance index; TI, tolerance index; SI, stress index; STS, stress tolerance score. Values were retained to two decimal places. Information on the genotypes corresponding to the serial numbers is given in Table S1. LP is low phosphorus stress, and D is PEG-induced drought stress; DLP is combined drought and low phosphorus stress.

Table S7. Broad-sense heritability (H) estimates for the 23 tested traits under the four different treatments (C, D, LP and DLP).

Trait	H_C	H_{LP}	H_D	H_{DLP}	H_{com}
**MRD**	0.47	0.23	0.30	0.52	0.58
**RN**	0.93	0.88	0.76	0.86	0.57
**RL**	0.67	0.59	0.36	0.66	0.54
**RD**	0.53	0.40	0.74	0.59	0.81
**RA**	0.41	0.52	0.21	0.35	0.55
**RV**	0.51	0.43	0.43	0.62	0.82
**RL_s1**	0.40	0.36	0.06	0.12	0.56
**RD_s1**	0.32	0.01	0.07	0.22	0.59
**RA_s1**	0.36	0.32	0.43	0.14	0.48
**RV_s1**	0.40	0.10	0.19	0.30	0.58
**RL_sub**	0.54	0.50	0.34	0.33	0.52
**RD_sub**	0.49	0.47	0.77	0.59	0.78
**RA_sub**	0.57	0.61	0.39	0.38	0.56
**RV_sub**	0.53	0.50	0.59	0.65	0.83
**RDW**	0.67	0.57	0.26	0.80	0.28
**RPC**	0.62	0.08	0.18	0.25	0.35
**RP**	0.07	0.10	0.05	0.31	0.06
**SDW**	0.82	0.59	0.71	0.63	0.46
**TDM**	0.85	0.66	0.66	0.79	0.46
**SH**	0.61	0.54	0.59	0.45	0.74
**LN**	0.93	0.78	0.87	0.70	0.55
**SPC**	0.41	0.25	0.12	0.18	0.25
**SP**	0.82	0.57	0.70	0.63	0.45

H denotes broad sense heritability under each treatment and H_{com} denotes combined heritability. Trait descriptions and units see Table 2. C is control treatment; LP is low phosphorus stress; and D is PEG-induced drought stress; DLP is combined drought and low phosphorus stress. MRD, maximum root depth; RN, root number; RL, root length; RD, root diameter; RA, root area; RV, root volume; RL_s1, root length s1; RD_s1, root diameter s1; RA_s1, root area s1; RV_s1, root volume s1; RL_sub, root length in sub layer; RD_sub, root diameter in sub layer; RA_sub, root area in sub layer; RV_sub, root volume in sub layer; RDW, root dry weight; RPC, root phosphorus concentration; RP, root phosphorus content; SDW, shoot dry weight; TDM, total dry mass; SH, shoot height; LN, leaf number; SPC, shoot phosphorus concentration; SP, shoot phosphorus content.

Table S8. Information on 100 winter wheat genotypes used in this study and descriptions of their stress tolerance scores based on this study.

No.	Name	Genotype origin	STS_{LP}	STS_{D}	STS_{DLP}	STS_{SUM}
**1**	Huazhang 166	Anhui Province	2.79	3.17	2.64	8.60
**2**	Fengdecunmai 21	Henan Province	3.37	3.71	3.13	10.21
**3**	Yongminmai No.1	Anhui Province	3.23	3.67	3.11	10.01
**4**	Pumai 116	Henan Province	3.29	3.85	2.99	10.13
**5**	Yongfeng103	Henan Province	3.12	3.30	2.90	9.32
**6**	Zhongzhimai No.13	Beijing	2.62	2.85	2.54	8.01
**7**	Xinong 979	Shaanxi Province	5.12	6.02	4.88	16.02
**8**	Fengdeicunmai 22	Henan Province	3.79	4.13	3.38	11.30
**9**	Hangyu 33	Huang-Huai Area	2.33	2.51	2.33	7.17
**10**	Saidemai No.8	Henan Province	3.50	3.99	3.19	10.68
**11**	Womai 33	Anhui Province	2.01	1.77	1.98	5.76
**12**	Deyan 0516	Huang-Huai Area	2.77	3.11	2.68	8.56
**13**	Baimai 312	Huang-Huai Area	3.17	3.53	2.93	9.63
**14**	Huaimai 40	Jiangsu Province	2.28	2.43	2.24	6.95
**15**	Zhengmai 16	Henan Province	2.25	2.15	2.18	6.58
**16**	Huaimai 226	Anhui Province	3.45	3.71	3.16	10.32
**17**	Xinong 556	Shaanxi Province	1.64	0.35	1.69	3.68
**18**	Lankao 298	Henan Province	2.55	2.80	2.39	7.74
**19**	Xinmai 2111	Henan Province	2.88	3.13	2.92	8.93
**20**	Hangyu 33	Huang-Huai Area	3.99	4.74	3.73	12.46
**21**	Bainong 418	Henan Province	2.70	3.03	2.60	8.33
**22**	Yongminmai No.1	Anhui Province	3.11	3.40	3.01	9.52
**23**	Zhengmai 518	Henan Province	2.59	2.75	2.44	7.78
**24**	Saidemai 601	Henan Province	3.25	3.60	3.13	9.98
**25**	Xinong 0615	Shaanxi Province	2.68	2.94	2.50	8.12
**26**	Huaimai 920	Jiangsu Province	3.88	4.33	3.90	12.11
**27**	Zhongliang 91250	Gansu Province	3.72	4.55	3.93	12.20
**28**	Shannong 7064	Shandong Province	3.65	4.27	3.78	11.70
**29**	Hefeng No.3	Henan Province	3.02	3.33	2.88	9.23
**30**	Shunmai No.11	Henan Province	2.91	3.25	2.74	8.90
**31**	Minfeng 266	Henan Province	2.31	2.24	2.23	6.78
**32**	Zhoumai 18	Henan Province	3.41	3.81	3.08	10.30
**33**	Xuke No.6	Henan Province	3.61	4.11	3.27	10.99
**34**	Cunmai 633	Guangdong Province	2.94	3.31	2.78	9.03
**35**	Bainong 558	Henan Province	4.03	4.57	3.77	12.37
**36**	Luomai 26	Henan Province	2.99	3.34	3.03	9.36
**37**	Ruiquanmai 32	Huang-Huai Area	2.72	3.08	2.64	8.44
**38**	Qinnong 168	Shaanxi Province	3.64	4.03	3.48	11.15
**39**	Tainong 33	Shandong Province	2.55	2.74	2.40	7.69
**40**	Lunxuan 2000	Hebei Province	3.30	3.57	3.06	9.93
**41**	Lankaoai 6	Henan Province	3.71	4.04	3.34	11.09
**42**	Yikemai No.5	Anhui Province	2.87	3.07	2.80	8.74
**43**	Huaihe 12148	Jiangsu Province	4.27	4.40	4.08	12.75

**44**	Bonong No.6	Henan Province	2.94	3.12	2.69	8.75
**45**	Xinmai 21	Henan Province	2.86	3.09	2.82	8.77
**46**	Yanzhan 4110	Henan Province	2.76	3.00	2.66	8.42
**47**	Xunong No.10	Henan Province	3.14	3.63	3.19	9.96
**48**	Tunmai 257	Henan Province	3.11	3.36	2.95	9.42
**49**	Xinong 501	Shaanxi Province	2.17	2.33	2.11	6.61
**50**	Longpingmai No.3	Anhui Province	3.62	3.82	3.33	10.77
**51**	Cunmai 30	Henan Province	2.78	3.16	2.56	8.50
**52**	Zhongmai 166	Henan Province	3.45	3.64	3.34	10.43
**53**	Huaimai 1403	Huang-Huai Area	2.73	3.00	2.56	8.29
**54**	Fumai 0808	Huang-Huai Area	3.05	3.46	2.98	9.49
**55**	Xinong 20	Shaanxi Province	2.71	2.99	2.70	8.40
**56**	Jimai 44	Shandong Province	3.58	3.77	3.16	10.51
**57**	Yunong 168	Henan Province	4.90	5.61	4.95	15.46
**58**	Baoliang No.5	Henan Province	3.19	3.83	3.02	10.04
**59**	Saidemai 601	Henan Province	3.71	4.44	3.69	11.84
**60**	Suyu 0622	Huang-Huai Area	2.21	2.28	2.18	6.67
**61**	Xiaoyan 22	Shaanxi Province	3.70	3.95	3.38	11.03
**62**	Baofeng 1530	Jiangsu Province	2.26	2.29	2.15	6.70
**63**	Shannong 116	Shandong Province	3.29	3.56	3.19	10.04
**64**	Pingan 0658	Henan Province	2.58	2.81	2.41	7.80
**65**	Qinnong 578	Shaanxi Province	2.34	2.57	2.32	7.23
**66**	Quanmai31	Henan Province	3.34	3.82	3.12	10.28
**67**	Saidemai No.2	Henan Province	2.66	2.93	2.53	8.12
**68**	Xinong 235	Shaanxi Province	2.98	3.59	2.87	9.44
**69**	Qinnong 28-6	Shaanxi Province	3.22	4.00	3.12	10.34
**70**	Zhongyu 1401	Henan Province	4.29	4.68	4.12	13.09
**71**	Gaoke 115	Henan Province	3.29	3.84	2.94	10.07
**72**	Luomai 906	Henan Province	2.82	3.18	2.85	8.85
**73**	Huacheng 5157	Anhui Province	3.88	4.19	3.62	11.69
**74**	Bainong 207	Henan Province	2.59	2.86	2.56	8.01
**75**	Huaihe 13068	Jiangsu Province	2.62	2.94	2.64	8.20
**76**	Taihemai No.6	Henan Province	2.87	3.30	2.62	8.79
**77**	Fengdecunmai 16	Henan Province	2.28	2.31	2.24	6.83
**78**	Xinzhi No.6	Henan Province	3.23	3.67	3.18	10.08
**79**	Shunmai No.10	Henan Province	2.64	2.83	2.53	8.00
**80**	Wunong 68	Hubei Province	2.65	2.87	2.56	8.08
**81**	Xunong 029	Jiangsu Province	3.33	3.91	3.27	10.51
**82**	Xuke 718	Henan Province	3.25	3.63	3.22	10.10
**83**	Jinxiu 21	Henan Province	2.70	3.00	2.56	8.26
**84**	Qinnong 29	Shaanxi Province	5.70	7.68	6.14	19.52
**85**	Yumai 64	Yunnan Province	3.91	4.71	3.92	12.54
**86**	Xinmai 38	Henan Province	2.88	3.26	2.81	8.95
**87**	Qinmai 618	Shaanxi Province	2.71	2.99	2.66	8.36
**88**	Longke 1221	Anhui Province	2.87	3.23	2.85	8.95
**89**	Bainong 419	Henan Province	3.47	4.18	3.84	11.49
**90**	Xiansheng 368	Henan Province	3.77	4.13	3.41	11.31

**91**	Zhongyuan 20	Henan Province	2.95	3.33	2.73	9.01
**92**	Huamai 304	Jiangsu Province	3.60	4.35	3.40	11.35
**93**	Tianmai 186	Henan Province	3.29	3.66	3.10	10.05
**94**	Zhongmai 99	Henan Province	3.22	3.50	3.04	9.76
**95**	Zhongzhongmai 27	Huang-Huai Area	2.87	3.23	2.94	9.04
**96**	Yanfeng 168	Henan Province	3.17	3.67	3.19	10.03
**97**	Huaimai 1196	Jiangsu Province	3.18	3.81	2.94	9.93
**98**	Zhongzhongmai 18	Huang-Huai Area	2.37	2.57	2.36	7.30
**99**	Zhongzhimai No.13	Huang-Huai Area	2.46	2.56	2.35	7.37
**100**	Zhengmai 9023	Henan Province	3.92	4.50	3.65	12.07

STS_{LP}, STS_{D}, and STS_{DLP} denote stress tolerance scores of winter wheat genotype under low-phosphorus stress (LP), PEG-induced drought stress (D), and combined drought and low phosphorus stress (DLP) treatments, respectively; STS_{SUM} denotes the total stress tolerance score from this study.
